# Cell Proliferation and Collective Cell Migration During Zebrafish Lateral Line System Development Are Regulated by Ncam/Fgf-Receptor Interactions

**DOI:** 10.3389/fcell.2020.591011

**Published:** 2021-01-14

**Authors:** Ramona Dries, Annemarie Lange, Sebastian Heiny, Katja I. Berghaus, Martin Bastmeyer, Joachim Bentrop

**Affiliations:** Zoological Institute, Cell- and Neurobiology, Karlsruhe Institute of Technology (KIT), Karlsruhe, Germany

**Keywords:** NCAM, proliferation, zebrafish, lateral line system, developmental biology, FGFR

## Abstract

The posterior lateral line system (pLLS) of aquatic animals comprises small clustered mechanosensory organs along the side of the animal. They develop from proneuromasts, which are deposited from a migratory primordium on its way to the tip of the tail. We here show, that the Neural Cell Adhesion Molecule Ncam1b is an integral part of the pathways initiating and regulating the development of the pLLS in zebrafish. We find that morpholino-knockdowns of *ncam1b* (i) reduce cell proliferation within the primordium, (ii) reduce the expression of Fgf target gene *erm*, (iii) severely affect proneuromast formation, and (iv) affect primordium migration. Ncam1b directly interacts with Fgf receptor Fgfr1a, and a knockdown of *fgfr1a* causes similar phenotypic changes as observed in *ncam1b*-morphants. We conclude that Ncam1b is involved in activating proliferation by triggering the expression of *erm*. In addition, we demonstrate that Ncam1b is required for the expression of chemokine receptor Cxcr7b, which is crucial for directed primordial migration. Finally, we show that the knockdown of *ncam1b* destabilizes proneuromasts, suggesting a further function of Ncam1b in strengthening the cohesion of proneuromast cells.

## Introduction

The zebrafish posterior lateral line system (pLLS) is a unique model for studying collective cell migration as well as axonal outgrowth and pathfinding during development of a sensory organ (Metcalfe et al., 1985; Kimmel et al., 1995; Gilmour et al., 2002; Dambly-Chaudière et al., 2007; Aman and Piotrowski, 2008; Lush and Piotrowski, 2014; Romero-Carvajal et al., 2015). Its development starts with the formation of the posterior primary primordium (PrimI), which arises from the disintegration of the posterior primary sensory placode (Gompel et al., 2001; Nikaido et al., 2017). PrimI migrates along the horizontal myoseptum and deposits up to 6 proneuromasts before reaching the tip of the tail at 48 hpf (Gompel et al., 2001; Lecaudey et al., 2008). Deposited proneuromasts differentiate into neuromasts, the sensory organs of the pLLS. Cell-cell interactions in the primordium initiate three major events of PrimI development: (i) Cell proliferation on the one hand takes place in the posterior, leading part (Leading-Zone) where it is driven by expression of the Wnt target gene *lef1* (Aman et al., 2011). On the other hand, Fgfr1a signaling in the anterior, trailing part (Trailing-Zone) has been identified as a key player for primordial proliferation (Aman et al., 2011). (ii) Directional collective cell migration is a consequence of the polarized expression of Cxcr4b and Cxcr7b in PrimI. These chemokine receptors enable PrimI to generate and sense a gradient of chemokine ligand Cxcl12a/Sdf1 which initially is uniformly expressed along the horizontal myoseptum (Knaut et al., 2003; Valentin et al., 2007; Boldajipour et al., 2008; Donà et al., 2013; Venkiteswaran et al., 2013; Nogare et al., 2014; Lau et al., 2020). Collective migration is mediated by cadherin-dependent adhesion between primordial cells (Colak-Champollion et al., 2019). (iii) Proneuromast formation in the Trailing-Zone is initiated by Fgf signaling. Under the influence of Fgf apically constricted rosettes of cells are established once a group of cells has evaded the influence of Wnt signaling in the Leading-Zone (Aman and Piotrowski, 2008; Lecaudey et al., 2008; Nechiporuk and Raible, 2008). Furthermore, Fgf activation initiates the specification of the centrally located hair cell, the first sensory cell of neuromasts (Lecaudey et al., 2008; Nechiporuk and Raible, 2008).

As we have discovered that Neural Cell Adhesion Molecule 1 (Ncam1) is expressed in the zebrafish pLLS (Langhauser et al., 2012), we intended to investigate its function during pLLS development. NCAM1 is a well-characterized glycoprotein of the immunoglobulin superfamily (IgSF). It is involved in regulating cell adhesion, cell proliferation and cell migration as well as neuritogenesis and plasticity in the adult nervous system (Hinsby et al., 2004). Consisting of five extracellular immunoglobulin-like (Ig) and two fibronectin (FN) domains, the extracellular part of NCAM1 can be involved in numerous homophilic and heterophilic interactions (Cunningham et al., 1987). Besides binding to various members of the IgSF mammalian NCAM1 displays a high binding affinity to Fibroblast Growth Factor Receptor 1 (FGFR1) (Saffell et al., 1994; Williams et al., 1994a,b,c). Interactions between NCAM1 and FGFR1 can occur within the same membrane (*cis*) and/or between two cells (*trans*) (Saffell et al., 1994; Williams et al., 1994a; Kiselyov et al., 2003; Francavilla et al., 2009; Christensen et al., 2011; Zamai et al., 2019). Like NCAM1, FGFR1 comprises three extracellular Ig-like domains with several ligand recognition sites (Johnson and Williams, 1993). The intracellular part of FGFR1 contains two tyrosine kinase domains with up to seven tyrosine residues (Beenken and Mohammadi, 2009).

Binding sites for interactions of NCAM1 and FGFR1 have been identified in the extracellular domains of the molecules (Williams et al., 1994a; Kiselyov et al., 2003; Christensen et al., 2011). Alternative splicing leads to the expression of two isoforms of FGFR1 (FGFR1-IIIb and –IIIc) which vary in the 3rd Ig-domain (Itoh and Ornitz, 2004; Li et al., 2010; Christensen et al., 2011) and bind to NCAM1 with different affinity. Upon binding, FGFR1 can activate various downstream signaling pathways. On the one hand, interaction with NCAM1 triggers the canonical FGFR1 signaling cascades activating PLCγ, PI3-Akt, or Ras-MAPK; it thereby promotes calcium influx and gene expression (Williams et al., 1992; Neiiendam et al., 2004; Anderson et al., 2005; Francavilla et al., 2009; Zamai et al., 2019). On the other hand, NCAM1 can elicit a FGFR1-mediated cellular response remarkably different from that initiated by binding of the FGF ligand. It induces the formation of FGFR1 complexes, which trigger Erk phosphorylation in a Src-dependent manner, whereas FGF binding triggers Erk phosphorylation via Ras (Zamai et al., 2019).

Due to a genome duplication in the teleost lineage, the zebrafish expresses two Ncam1 paralogs: Ncam1a and Ncam1b. These paralogs have subfunctionalized roles in embryonic development, as we have previously shown for the formation of the posterior commissure and for outgrowth, fasciculation and pathfinding of spinal motor axon bundles (Langhauser et al., 2012). In this study, we asked if Ncam1a and Ncam1b are also involved in the development of the zebrafish posterior lateral line system. We find that a knockdown of *ncam1b* severely affects the morphogenesis and the migration of PrimI. Ncam1b directly interacts with Fgfr1a, and a knockdown of *fgfr1a* causes similar phenotypic changes as observed in *ncam1b*-morphants. We identify Ncam1b as a novel factor involved in activating proliferation in the primordium, probably by triggering the expression of the Fgfr1a target gene *erm*. Finally, we show that Ncam1b is required for the expression of *cxcr7b*, which uncovers an as yet unknown function of Ncam1b in the directed migration of PrimI during pLLS development.

## Materials and Methods

### Fish Strains and Animal Care

In all experiments we used the transgenic fish strain *Tg(ClaudinB::lynGFP)*, which was kindly given to us by Darren Gilmour (University of Zürich, CH) (Haas and Gilmour, 2006). Zebrafish were maintained at 28.5°C under standard conditions in a ZebTec system (Tecniplast; Buguggiate, Italy). Embryos were kept in standard embryo medium (E3) at 28.5°C for normal development. To obtain embryos at 36 hpf (hours post fertilization), eggs were incubated for 24 h at 28.5°C and for another 24 h at 25°C. Embryos were manually dechorionated with forceps after 24 hpf. Staging refers to Kimmel et al. (1995).

### Morpholino and mRNA Injection

Morpholino or mRNA were pressure-injected into the yolk at the 1-cell stage. Morpholinos (Gene Tools, Oregon, USA) and mRNA (Langhauser et al., 2012) were used in the following concentrations per embryo: *standard control*-Mo (5′-CCTCTTACCTCAGTTACAATTTATA-3′), same as that of the relevant knockdown-Mo; *ncam1b*-ATG-Mo (5′-AGATTATCGCCTTGGTCGGAAACAT-3′), 3 ng; *ncam1b*-ATG-5 mismatches-Mo (5′-ACATTATGGCCTTCGTCGCAAAGAT-3′), 3 ng; *ncam1b*-5′UTR-Mo (5′-GTTTACTGTTTGTTTTTGCCTTCCG-3′), 3 ng; p53-Mo (5′-GCGCCATTGCTTTGCAAGAATTG-3′), 3 ng; *ncam1a*-5′UTR-Mo (5′-TTCCGTGTAGAATAGGTAGAGTTGG-3′), 3 ng; *erm*-Mo (5′-GAGAAGCAAGCGACATGGATGGGTT-3′), 5 ng; *fgfr1a*-Mo (5′-GCAGCAGCGTGGTCTTCATTATCAT-3′), 12 ng for 24 hpf and 18 ng for 48 hpf; *ncam1b*-mRNA, 0.3 ng (Langhauser et al., 2012).

### Time-Lapse Imaging

Time-lapse imaging was performed beginning at 28 hpf in standard embryo medium (E3). Embryos were dechorionated with forceps, anesthetized in 0.01% MS-222 solution and finally embedded in 1.5% low melt agarose. Agarose was covered with a thin film of embryo medium containing 0.01% MS-222 to avoid evaporation. Images were taken every 15 min for a total duration of 17 h (1,020 min). Imaging was performed on a Zeiss Axiovert 200M with 10× magnification.

### Immunostaining Zebrafish

Immunostaining was performed following zebrafish standard procedures as previously described (Marx et al., 2007). Ncam1a and Ncam1b were detected by rabbit anti-NCAM and anti-PCAM kindly provided by Yoshihiro Yoshihara (RIKEN Center for Brain Science, Japan) (both 1:1,000) and Zo-1 was detected by mouse Zo-1 (1:200; Fisher Scientific; Massachusetts, USA). After *in situ* hybridization, GFP intensity in *Tg(ClaudinB::lynGFP)* embryos was enhanced by using rabbit anti-GFP (1:1,000; abcam; Cambridge, GB), as indicated in the figure legends.

Embryos were subsequently placed in secondary antibody for 2 h at room temperature. For secondary antibody staining, Alexa488 (1:1,000; Molecular Probes; Oregon, USA) and cy3 (1:1,000; Dianova; Hamburg, Germany) were used as chromophors. Mounting was performed in Mowiol. Images were taken with ZEISS LSM510 and LSM800.

### Whole-Mount *in situ* Hybridization

Whole-mount *in situ* hybridization was performed with DIG-labeled probes as described in Schulte-Merker et al. (1992) and Thisse and Thisse (2008). Probes were synthesized from plasmid DNA. For hybridization 2 ng/μl of each probe were used. Plasmid for *erm* (Scholpp et al., 2004) and *lef1* were kindly provided by Steffen Scholpp (University of Exeter, GB); *cxcr7b* (Dambly-Chaudière et al., 2007); and *cxcl12a* (Li et al., 2004) were kindly given by the lab of Tatjana Piotrowski (Stowers Institute, USA). Probe templates for *cxcr4b* (*forward*: 5′-ATGGAATTTTACGATAGCAT-3′; *reverse*: 5′-CTAACTCGTCAGTGCACTGG-3′) and *fgfr1a* (*forward*: 5′-TCAGATGTAGAGGATCTTTGATA-3′; *reverse*: 5′- ACACACACACTGAGTAAATGAGT-3′) were isolated and cloned from zebrafish cDNA. Afterwards embryos were stained with anti-GFP (1:1,000; abcam; Cambridge, GB) and DAPI (1:1,000; Carl Roth; Karlsruhe, Germany) and were embedded in Mowiol.

### Western Blot Analysis

Siblings and morpholino injected embryos were deyolked and lysed in 1× SDS buffer. After heating and sonification cell lysates were cleared by centrifugation at 13,000 g for 5 min. Proteins (five embryos/lane) were resolved by SDS-PAGE and transferred to PVDF membranes. For protein detection antibodies against p53 (mouse; 1:250; abcam; Cambridge, GB), Ncam1b [rabbit; 1:4,000; Yoshihiro Yoshihara (RIKEN Center for Brain Science, Japan)] and acetylated tubulin (mouse; 1:4,000; Sigma-Aldrich; Missouri, USA) were used, followed by specific peroxidase-conjugated secondary antibodies (1:10,000). Super Signal West Pico PLUS Chemiluminescent Substrate (Fisher Scientific; Massachusetts, USA) was used for chemiluminescent visualization.

### BrdU Assay

Embryos were chilled on ice in E3 medium for 15 min, followed by incorporation of 10 mM BrdU (Sigma-Aldrich; Missouri, USA) for 20 min on ice. Afterwards embryos were washed several times with pre-warmed E3 medium and were finally fixed with 4% PFA in PBS overnight at 4°C. Fixed embryos were stored in methanol at −20°C overnight. Permeabilization was performed by ProteinaseK treatment (10 μg/ml) for 10 min at room temperature, followed by a PBST wash step. Samples were treated with 2 N HCl for 1 h at 37°C. Finally, embryos were blocked in 1% BSA and incubated overnight with anti-BrdU (mouse; 1:100; abcam; Cambridge, GB) and anti-GFP (rabbit; 1:1,000; abcam; Cambridge, GB) at 4°C. On the next day embryos were washed with PBST and subsequently stained with secondary antibody [Alexa488; 1:1,000; (Molecular Probes; Oregon, USA), cy3; 1:1,000; (Jackson Immunoresearch; Pennsylvania, USA)] and DAPI (1:1,000; Carl Roth; Karlsruhe, Germany). Mounting was performed in Mowiol. For quantification BrdU-DAPI-positive cells within the primordium of all embryos were counted at a ZEISS LSM510.

### Isolation of Fgfr1a-Isoforms and Expression Vectors

Fgfr1a isoforms were isolated from total zebrafish mRNA at different developmental stages. RNA was isolated by TriFAST (peqlab; Erlangen, Germany), mRNA was converted into cDNA by reverse transcription (Fisher Scientific; Massachusetts, USA) using Fgfr1a-specific primers (*forward*: 5′-TCAGATGTAGAGGATCTTTGATA-3′; *reverse*: 5′-ACTCATTTACTCAGTGTGTGTGT-3′). After amplification blunt-ended DNA was cloned into pCR-BluntII-TOPO (Thermo Scientific, Pittsburgh, USA).

For the expression of membrane-bound proteins, full coding sequences were cloned into the expression vector pcDNA 3.1 with Myc- and 6× His-Tag (Invitrogen; California, USA). *fgfr1a-w/o exon 7* was synthesized by Fusion-PCR excluding exon 7. The coding sequences for soluble proteins, which comprised only the extracellular parts of Ncam1 or Fgfr1a, were cloned into pcDNA 3.1 with hIgG-Fc-Tag (Rita Gerardy-Schahn, Hannover Medical School, Germany).

### Cell Lines, Transfection, and Protein Purification

Overlay assays were performed using *CHO-K1* cells, which were cultured in DMEM with 10% FCS at 37°C in a humidified incubator. Cells were transfected with Lipofectamine2000 (Invitrogen; California, USA) according to the manufacturer′s instructions. Selection occured by antibiotic treatment with geneticin (750 μg/ml).

*CHO-2A10* cells were used for expression of soluble proteins. To that end, transfected cells were incubated for 12 days in DMEM with 10% FCS and were selected by adding zeocin (750 μg/ml). After selection, cells were incubated for at least 4 weeks in OPTI-CHO medium containing 4 mM L-Glutamine. Medium was collected by decanting and centrifugation; the resulting supernatant was stored at 4°C after adding 0.02% Thimerosal (Sigma-Aldrich; Missouri, USA) and Complete Protease Inhibitor (Roche; Basel, Switzerland). Medium was concentrated by Vivaflow2000 (Sartorius; Göttingen, Germany) and Fc-Tagged protein was captured by HiTrap ProteinG HP (GE Healthcare; Illinois, USA). Finally, the eluted protein was concentrated by Amicon Ultra (Merck; Darmstadt, Germany).

### Overlay Assay and Immunostaining Cell Culture

*CHO-K1* cells were transfected with expression vector pcDNA 3.1 Myc and 6× His-Tag containing complete coding sequence of either *ncam1a, ncam1b, fgfr1a-IIIb, fgfr1a-IIIc*, or *fgfr1a-w/o exon 7*. After antibiotic selection cells were seeded on fibronectin-coated coverslips (10 μg/ml) and cultured overnight. The next day, coverslips were incubated with 20 μg/ml of soluble protein for 1 h at room temperature. Unbound protein was removed by several wash steps. Cells were subsequently fixed with 4% PFA in PBS and treated with sodium borohydride to reduce double bounds. Permeabilization was performed with 0.1% PBST. Incubation with primary antibody anti-His (mouse; 1:400; abcam; Cambridge, GB) and anti-hFcγ (goat; 1:400; Jackson Immunoresearch; Pennsylvania, USA) was performed for 1 h at room temperature. After washing with PBS cells were incubated with secondary antibody [Alexa488 (1:1,000; Molecular Probes; Oregon, USA) and cy3 (1:1,000; Dianova; Hamburg, Germany)], DAPI (2 μg/ml; Carl Roth; Karlsruhe, Germany) and Phalloidin (13 U/ml; Life Technologies; California, USA). Images were taken with ZEISS Axio Imager.Z1 in the Apotome mode. To quantify the difference of Ncam1a/b binding to FGFR-transfected cells vs. the binding to untransfected cells, we first evaluated the efficiency of transfection of membrane-bound, His-tagged Fgfr1a using Fiji by ImageJ. Based on these data we calculated the ratio of the fluorescence intensities resulting from the binding of soluble Fc-tagged Ncam1a/b to transfected vs. non-transfected cells.

### Co-immunoprecipitation

*Hek293* cells were co-transfected with expression vector pcDNA3.1 Myc and 6× His-Tag containing complete coding sequences of *ncam1a* or *ncam1b*, respectively, and with expression vector pcDNA3.1 FLAG-Tag containing the complete coding sequence of *fgfr1a-IIIb* or *fgfr1a-IIIc*, respectively. Single transfections served as a control; to that end *Hek293* cells were transfected either with expression vector pcDNA3.1 Myc and 6× His-Tag containing the complete coding sequence of *ncam1b* or with expression vector pcDNA3.1 FLAG-Tag containing the complete coding sequence of *fgfr1a-IIIb*. After antibiotic selection cells were lysed in lysis buffer (Cell Signaling Technology, Massachusetts, USA). Cell lysates were incubated with an anti-His-antibody (rabbit; 1:100; abcam; Cambridge, GB) overnight. ProteinA beads (Cell Signaling Technology, Massachusetts, USA) were added at a final dilution of 1:10 and incubated by gently shaking at 4°C for 1.5 h. SDS-PAGE and Western Blot were performed following a modified *Laemmli* protocol (Laemmli, 1970). Each loaded sample contains the material of 100,000 cells. After SDS-PAGE, immunoprecipitates were immunoblotted with biotinylated FLAG-antibody (mouse; 1:800; Sigma-Aldrich; Missouri, USA), followed by HRP-conjugated streptavidin (1:6,000; Sigma-Aldrich; Missouri, USA). Band density (Intensity Density) was measured with Fiji by ImageJ for three independent experiments.

### Bead Aggregation Assay

Bead aggregation assays were performed as described (Galuska et al., 2010). Fluorescent beads are pre-coated with ProteinA (Kisker Biotech; Steinfurt, Germany) and then incubated with 10 μg Fc-Tagged soluble protein of either Ncam1a, Ncam1b, Fgfr1a-IIIb, or Fgfr1a-IIIc. Controls were performed with soluble human Fc-fragment (Merck; Darmstadt, Germany). Formation of bead aggregation was observed after 24 h in uncoated γ-slides (ibidi; Gräfelfing, Germany) with ZEISS LSM510.

### Sequence Analysis

Zebrafish sequences of Ncam1a, Ncam1b and Fgfr1a were compared with several mammalian and non-mammalian organisms by using CLC Sequence Viewer 8.0 (Qiagen Bioinformatics). For Ncam1 alignment (accession numbers are given in parentheses) the following sequences were used: *H. sapiens* (AAH47244.1), *M. musculus* (XP_006510118.1), *R. norvegicus* (XP_008764414.1), *G. gallus* (XP_015153520.1), *X. laevis* N1A (XP_018082333.1), *X. laevis* N1b (XP_018079824.1), *O. latipes* (XP_023818322.1), *Danio rerio* N1a (HM467818.1) and *Danio rerio* N1b (XP_005157462.1). For Fgfr1 alignment the subsequent sequences were used: *H. sapiens* (XP_006716366.1), *M. musculus* (AAA37620.1), *R. norvegicus* (NP_077060.1), *G. gallus* (XP_015152849.2), *X. laevis* (NP_001090457.1), *O. latipes* (XP_011477376.1), *Danio rerio* (IIIb-exon9(SS)) (MW358033), *Danio rerio* (IIIb-exon9(VT)) (MW358034) and *Danio rerio* (IIIc) (MW358035).

### Quantification and Statistical Analysis

**Figure 1:**
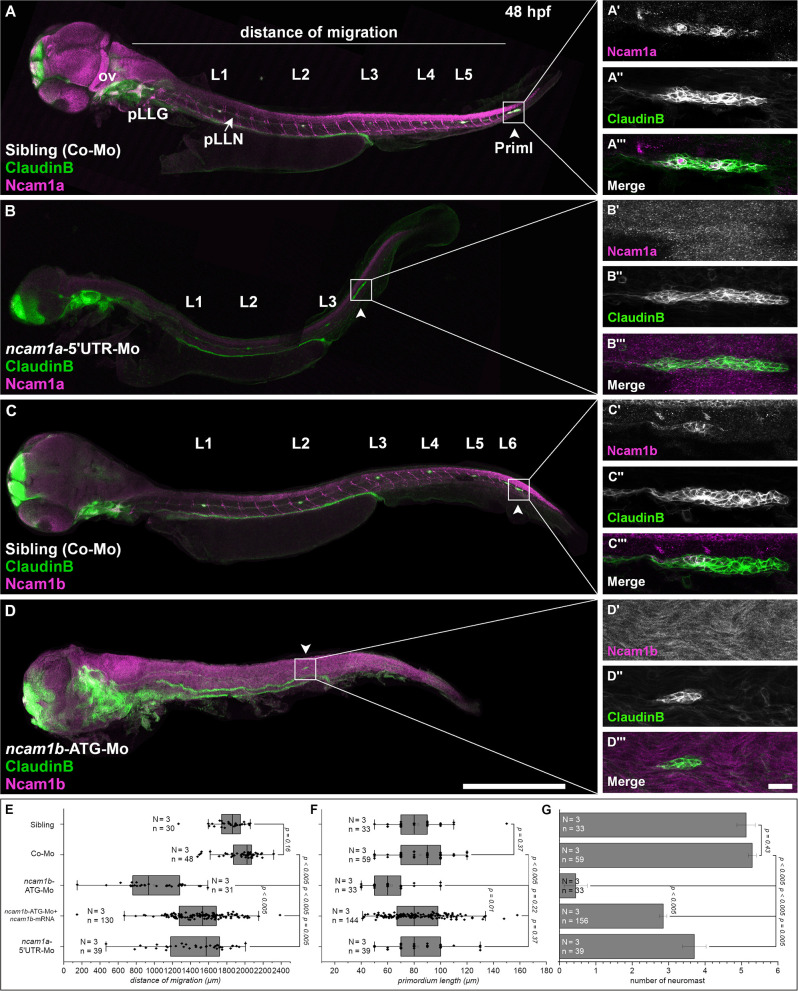
Expression pattern and function of Ncam1 during development of the zebrafish lateral line system. **(A–D)** Lateral views of *Tg(ClaudinB::lynGFP)* (green) embryos (48 hpf) immunostained for Ncam1a or Ncam1b (magenta). **(A,C)** Control-Morpholino (Co-Mo) injected sibling; all structures (excluding interneuromast cells) of the lateral line system express Ncam1a and Ncam1b. (**A′-A‴,C′-C‴**) Ncam1a is expressed throughout the whole primordium (arrowhead), whereas Ncam1b is found only in the Trailing-Zone. **(B)** *ncam1a*-morphants show only weak migration defects and (**B′-B‴**) primordium length is not affected. (**D**) Morpholino-knockdown of *ncam1b* induces migration defects as well as a reduction in the number of deposited (pro)neuromasts. **(D′-D‴)** *ncam1b-*knockdown also results in a reduced primordium size. (**E,F,G**) Quantification of migration distance, primordium length and number of neuromasts in control and morpholino-injected embryos. Rescue mRNA was co-injected with the morpholino as indicated. Error bars represent standard deviation. Scale bars **(A,D)** 200 μm, **(D‴)** 20 μm. Abbreviations: Co-Mo, Control-Morpholino; L1-6, lateral (pro)neuromast; pLLG, lateral line ganglion; pLLN, lateral line nerve; ov, otic vesicle; PrimI, primary posterior primordium.

For measurements of primordia size and migration distance we used Fiji by ImageJ. For quantification of knockdown phenotypes in Figure 1, only those embryos where considered, which did not show expression of the specific protein as judged by immunostaining. For quantification in Supplementary Figure S1 as well as in **Figure 5** and Supplementary Figure S8 all embedded embryos were imaged and analyzed. Statistical analysis was done by Origin2019. Boxplots show the 25th and 75th percentiles with median (line inside the box) and mean value (square). The whiskers extend to the minimum and the maximum. Outlier are marked by black diamonds. Bar charts show mean values. For significance calculation we used Students *t*-test or X^2^ analysis.

## Results

### Ncam1 Paralogs Are Expressed in the Posterior Lateral Line System (pLLS)

The posterior lateral line primordium starts its migration around 24 hpf. It follows the horizontal myoseptum for the next 24 hours until it reaches the tip of the embryo′s tail (Supplementary Videos 1, 2). During migration the primordium deposits proneuromasts, clusters of about 30 cells which subsequently differentiate into mature neuromasts. As cell adhesion and cell communication play crucial roles during the collective and directional cell migration and for proneuromast deposition, we investigated the role of neural cell adhesion molecule Ncam1 in this process. We find, that both zebrafish paralogs of Ncam1, namely Ncam1a and Ncam1b, are expressed during pLLS development (Figures 1A,C). Both are detectable in the lateral line ganglion (pLLG), the lateral line nerve (pLLN), neuromasts (L1-L6) as well as in the primordium. Notably, whereas Ncam1a is expressed by all cells of the primordium (Figures 1A′–A‴), Ncam1b is expressed only in the Trailing-Zone, but not in the Leading-Zone (Figures 1C′–C‴).

### Ncam1b Is Necessary for Correct pLLS Development

To study the functions of both paralogs we performed morpholino-knockdowns. We find that a knockdown of *ncam1a* significantly affects pLLS development (Figures 1B–B‴ and Supplementary Video 3) by reducing migration distance of the primordium and the number of deposited proneuromasts. The length of the primordium (PrimI) is not affected (Figures 1E–G). Knocking down *ncam1b* by using a morpholino that targets the ATG start codon region (Langhauser et al., 2012) causes significantly stronger phenotypic alterations of the developing pLLS (Figures 1D–D‴). Primordium length is drastically reduced (Figures 1D′–D‴,F) and the formation and deposition of proneuromasts are rarely observed (Figure 1G). Life imaging shows that only in some cases a single proneuromast is formed and deposited which then disintegrates rapidly (Supplementary Video 4). Migration of the primordium is also strongly affected (Figure 1E). In control-morpholino injected siblings the primordium reaches the tip of the tail at 48 hpf, whereas in *ncam1b*-morphants it reaches the end of the yolk extension at most (Figure 1D, Supplementary Video 4). In some cases, primordia deviate from their path along the horizontal myoseptum (see below). An increased background staining in immunohistochemistry after morpholino-knockdown of *ncam1b* is an unfortunate feature of the anti-PCAM antibody (see also Langhauser et al., 2012). This staining is unspecific, as the reduction of Ncam1b after morpholino injection is obvious in proven Ncam1b expression domains like the spinal cord, spinal motor axons and the primordium (compare Figures 1C,D,C′,D′; see also Western Blots in Supplementary Figure S1D). Co-injection of *ncam1b*-mRNA significantly mitigates the knockdown phenotype (Figures 1E–G).

Efficacy and paralog specificity of the used *ncam1b*-ATG-morpholino was already demonstrated by Langhauser et al. (2012) and we performed extensive additional control experiments to rule out potential off-target effects and to underpin the effectiveness of the morpholino knockdown. Injection of a second, non-overlapping 5′UTR-morpholino shows the same effects as the start codon morpholino (Supplementary Figures S1, S2). Phenotypes after injection of an unrelated control-morpholino and a mismatch-morpholino are indistinguishable from those of uninjected siblings (Supplementary Figures S1A–C). We rule out, that the reduced primordium size results from increased apoptosis, as co-injection of a p53-morpholino does not rescue the morpholino effect (Supplementary Figures S1A–C) and as we do not find an increase of p53 expression following morpholino injections (Supplementary Figure S1D). We find Ncam1b protein expression drastically reduced after injection of the start codon and the 5′UTR-morpholino, which is not the case after injection of control morpholinos (Supplementary Figure S1D).

### Proliferation of Primordial Cells Is Reduced in *ncam1b*-Morphants

**Figure 2:**
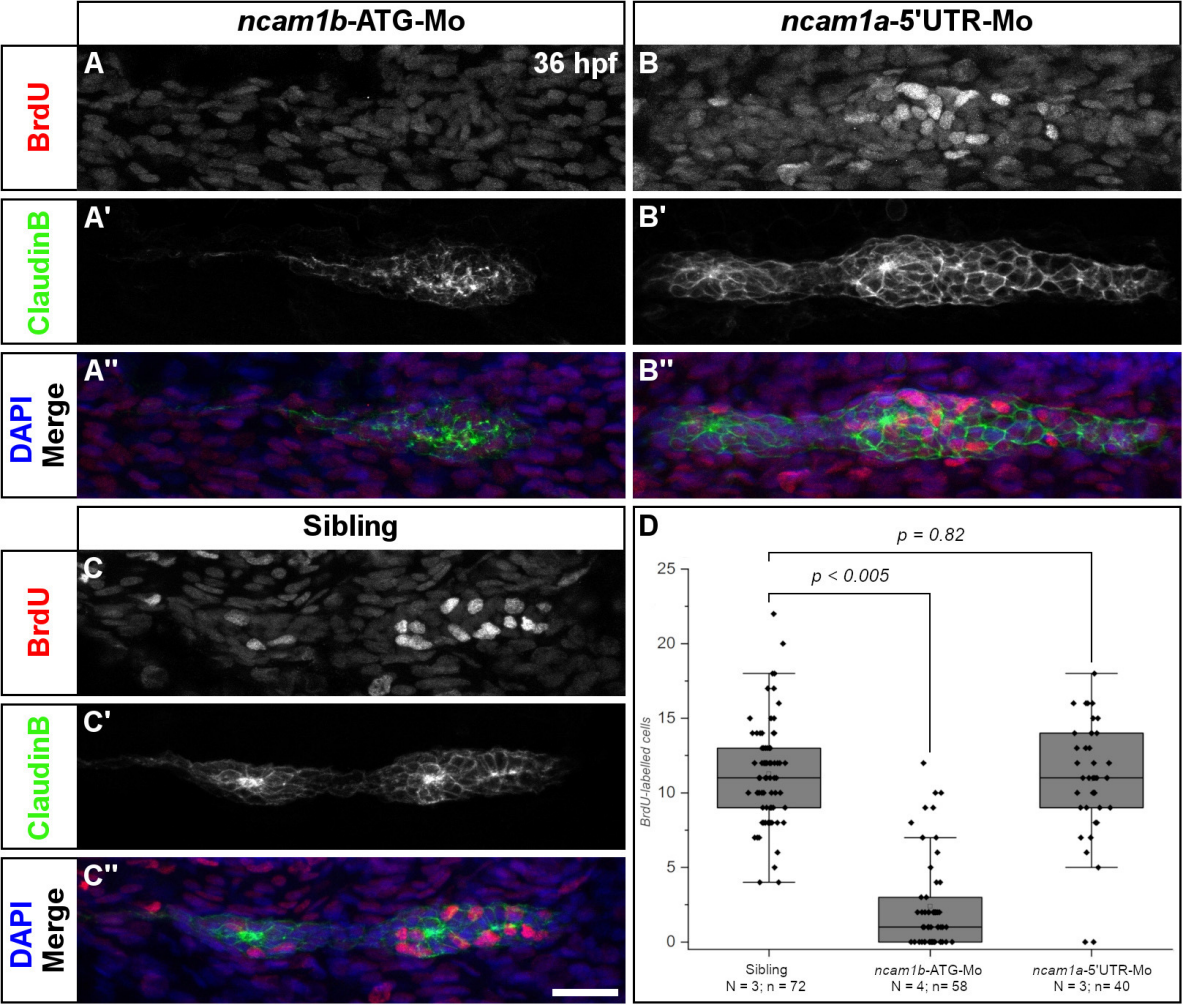
Ncam1b is required for cell proliferation within the primordium. Siblings or morpholino-injected *Tg(ClaudinB::lynGFP)* embryos were treated with BrdU at 36 hpf for 20 min, fixed and immunostained for BrdU (red), GFP (green) and DAPI (blue) as indicated. **(A,A″)** BrdU incorporation is almost absent in *ncam1b*-morphants, but obvious in **(B,B″)** knockdowns of *ncam1a* and **(C,C″)** uninjected siblings. **(D)** Quantification of BrdU-labeled cells within primordia. Scale bar 20 μm.

A reduction of primordia size and neuromast number in *ncam1b*-morphants could be explained by a reduced cell proliferation rate. Therefore, we performed BrdU incorporation assays. Embryos were treated with BrdU for 20 min and fixed. Cell proliferation in the primordia of 36 hpf uninjected siblings is detected in both Leading- and Trailing-Zones (Figures 2C–C″). Proliferation is severely decreased in *ncam1b*-morphants (Figures 2A–A″), which show a reduction of the number of BrdU-labeled cells by more than 90% (Figure 2D). A knockdown of *ncam1a* has no effect on cell division (Figures 2B–B″). These experiments indicate that Ncam1b is involved in primordial cell proliferation. It thereby affects primordium size as well as the formation of proneuromasts.

### Ncam1b Drives Expression of Fgfr1a-Target Gene *erm*

**Figure 3:**
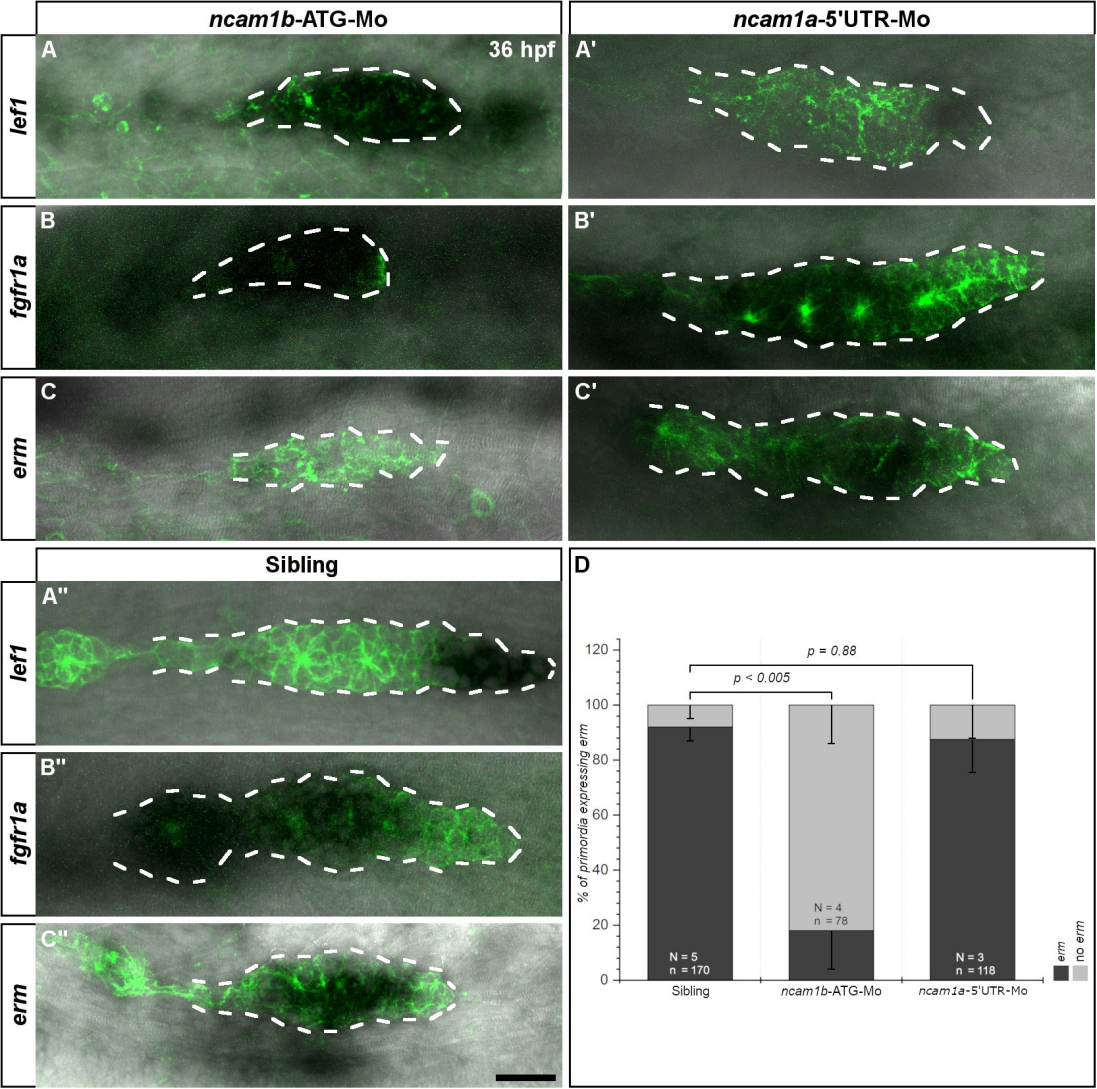
Ncam1b affects the expression of the Fgfr1a-target gene *erm*. *Tg(ClaudinB::lynGFP)* embryos were immunostained labeled for GFP (green). *In situ* hybridizations of probes detecting *lef1* (Leading-Zone), *fgfr1a*, or *erm* (both in the Trailing-Zone) were visualized by NBT/BCIP staining. Primordia outlines are surrounded with a dotted line for better visibility. **(A–C)** Neither expression of *lef1* nor of *fgfr1a* are affected in *ncam1b*-morphants, whereas *erm* expression is markedly reduced. (**A′,C′**) Neither *ncam1a*-morphants nor (**A″,C″**) siblings show alterations for *lef1, fgfr1a*, or *erm*. **(D)** Reduced *erm* expression after knockdown of *ncam1b*. Error bars show standard deviation. X^2^-test with *ns* = *p* ≥ *0.05*. Scale bar 20 μm.

It has been shown that primordial cell proliferation is mostly controlled by the Wnt-target gene *lef1* (Lecaudey et al., 2008; Gamba et al., 2010; Valdivia et al., 2011; Breau et al., 2013; Agarwala et al., 2015). We therefore analyzed *lef1* expression in *ncam1a-* and *ncam1b*-morphants by *in situ* hybridization. In 36 hpf uninjected siblings and *ncam1a*-morphants we detect *lef1* in the posterior one third of the primordium, which corresponds to the Leading-Zone (Figures 3A′,A″). Surprisingly, in *ncam1b*-knockdowns we observed an expanded *lef1* expression rather than a reduction (Figure 3A). While the knockdown primordium is somewhat smaller than its sibling counterpart, *erm* is expressed throughout the complete structure. Thus, Wnt signaling appears not to be suppressed in *ncam1b* morphants, which indicates that Wnt signaling pathways are not involved in the Ncam1b-dependent regulation of cell division.

Aman et al. (2011) had shown, that Wnt/β-catenin signaling acts in concert with signaling via the Fgf receptor Fgfr1a to promote cell division. Fgfr1a is expressed in the Trailing-Zone and an inhibition leads to a strong reduction in proliferation within the primordium (Aman et al., 2011). The relevant downstream signaling cascade has not yet been identified. We thus investigated Fgfr1a signaling by first analyzing the expression of *fgfr1a* by *in situ* hybridization. We could not detect differences in *fgfr1a* expression between uninjected siblings (Figure 3B″) and *ncam1a-*morphants (Figure 3B′). In *ncam1b*-morphants, despite an overall reduced primordium size, we still observe robust *fgfr1a* expression in the Trailing-Zone (Figure 3B). This implies that Leading*-* and Trailing-Zones are formed as distinct domains in *ncam1b*-morphants as well. Searching for a downstream target potentially involved in Fgfr1a-dependent proliferation, we next addressed the expression of the transcription factor Erm (Etv5b), which regulates proliferation and differentiation in mouse embryonic stem cells (Akagi et al., 2015). *In situ* hybridizations show *erm* expression within the primordial Trailing-Zone of 36 hpf uninjected siblings (Figures 3C′,C″) and *ncam1a*-morphants, where Fgfr1a is also expressed (Figures 3B′,B″). In *ncam1b*-morphants, however, we observed a strong reduction of *erm* within the primordia (Figures 3C,D). Other reported expression domains of *erm* (Roehl and Nüsslein-Volhard, 2001; Scholpp et al., 2004) like the midbrain-hindbrain boundary and the forebrain were not affected (Supplementary Figure S3). To test the role of Erm, we performed BrdU incorporation assays at 36 hpf after knockdown of *erm* (Supplementary Figure S4). Cell proliferation within the primordium is decreased, especially in the Trailing-Zone (Supplementary Figures S4A,C). These experiments suggest that Ncam1b and Fgfr1a, which are both expressed in the Trailing-Zone, act in the same pathway controlling cell proliferation by regulating the expression of *erm*.

### Different Splice Variants of Fgfr1a Are Expressed in the Zebrafish Embryo

**Figure 4:**
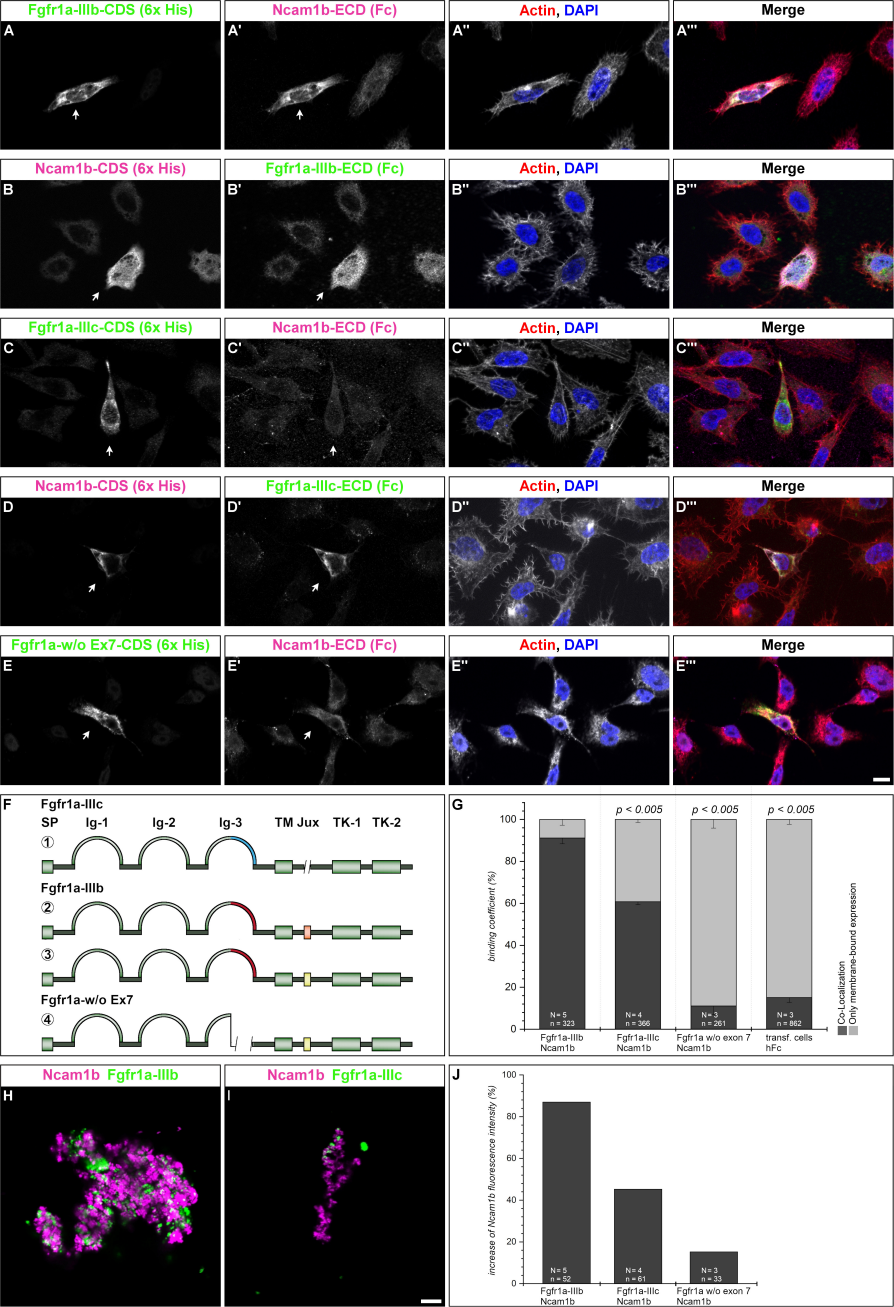
Ncam1b interactions with different isoforms of Fgfr1a. *CHO-K1* cells were transfected with complete coding sequences (CDS) of one of three Fgfr1a-isoforms (green; IIIb, IIIIc, or w/o Ex7) or of Ncam1b (magenta). Transfected cells were incubated for 1 hr with either soluble Ncam1b or one of the two Fgfr1a-isoforms, fixed and immunostained for His-, Fc-Tag, Actin (red), and DAPI (blue). (**A,B‴**) Ncam1b interacts strongly with Fgfr1a-IIIb irrespectively of which of the proteins is membrane-bound. (**C,D‴**) Binding of Ncam1b to Fgfr1a-IIIc is markedly weaker. (**E,E‴**) No interaction occurs if Fgfr1a lacks exon 7. (**F**) Scheme of isolated and cloned Fgfr1a splice variants: ① Fgfr1a-IIIc does not contain any exon 9, ② one splice variant of Fgfr1a-IIIb contains a juxtamembrane region of valine and threonine (orange), ③ another isoform of Fgfr1a-IIIb encodes two serine residues (yellow) within the juxtamembrane domain, ④ Fgfr1a-IIIa, an artificial variant of Fgfr1a, lacks the C-terminal part of Ig-3, which is encoded by exon 7. (**G**) Quantification of the binding of Ncam1b to splice variants of Fgfr1a. Binding coefficient represents the percentage of cells expressing the membrane-bound binding partner that were co-labeled by antibodies against the soluble binding partner. Error bars represent standard deviations (χ^2^-test). (**H,I**) Bead aggregation assays reveal a strong homophilic binding of Ncam1b. Ncam1b-coated beads interact with Fgfr1a-IIIb-coated beads and to a lower extend with Fgfr1a-IIIc. **(J)** Measuring the fluorescence intensity of soluble Ncam1b-Fc shows a strong binding to membrane-bound Fgfr1a-IIIb and a weaker to Fgfr1a-IIIc or Fgfr1a without exon 7. Scale bar in (**E‴**) represents 20 μm and in (**I**) 10 μm. Abbreviations: CDS, coding sequence; ECD, extracellular domain; lg, Immunoglobulin domain; Jux, juxtamembrane domain; SP, Signal peptide; TK, Tyrosine Kinase domain.

A direct interaction of NCAM1 with FGFR1 has already been observed in mammalian cell cultures, where it promotes neurite outgrowth (Williams et al., 1994a,b,c). Mammalian NCAM1 has different affinities for FGFR1 isoforms varying in the 3rd Ig-domain (Li et al., 2010; Christensen et al., 2011). To study an interaction of Ncam1b with Fgfr1a, we first searched for zebrafish isoforms of Fgfr1a that are expressed during pLLS development. From cDNA of 24 to 72 hpf embryos we isolated full length clones representing two isoforms, Fgfr1a-IIIb and –IIIc, which have previously been described by Scholpp et al. (2004). These isoforms differ by usage of different exons 7, which encode the C-terminal part of the 3rd Ig-domain. Whereas, exon 7.1 (blue in Figures 4F, 1) is included in *fgfr1a-IIIc* and has a size of 144 bp (48 aa), exon 7.2 (red in Figures 4F, 2, 3) is included in *fgfr1a-IIIb* and contains 147 bp (49 aa). Sequence analysis reveals a 47% homology among these alternative domains on the amino acid level. Further isoforms of the extracellular domain (ECD) were not discovered. Within the region encoding the intracellular juxtamembrane domain, *fgfr1a-IIIb* contains either exon 9.1 or exon 9.2, each of which codes for two amino acids (Figures 4F, 2, 3). Our interaction studies (see below) were performed only with the 9.1 variant of Fgfr1-IIIb since we assume that a two amino acid exchange in the intracellular region will not influence an interaction of Ncam1b and Fgfr1a. A zebrafish homolog of mouse Fgfr1a-IIIa, which lacks an exon 7-coded domain, could not be detected; we thus constructed this isoform artificially as a membrane-bound version (Figures 4F, 4).

### Ncam1b Interacts Differently With Fgfr1a Isoforms

To test for an interaction of Ncam1b with Fgfr1a, we performed overlay assays. Cells expressing a membrane-tethered, His-tagged version of one protein were incubated with the soluble, Fc-tagged extracellular portion of the potential interaction partner. For a first evaluation of a possible interaction, we calculated a binding coefficient. To that end we determined the percentage of cells expressing the membrane-bound ligand binding partner that were co-labeled by antibodies against the soluble binding partner. Cells expressing membrane-bound Fgfr1a-IIIb also show a strong labeling for soluble Ncam1b (Figures 4A–A‴, arrow) and vice versa (Figures 4B–B‴, arrow). Around 90% of the transfected cells show a co-localization of the soluble interaction partner (Figure 4G). The binding coefficient of Ncam1b to Fgfr1a-IIIc is about 60% (Figures 4C–C‴,D–D‴,G). The variant of Fgfr1a receptor lacking the exon 7 domain does not exceed binding coefficients above background levels, i.e., coefficients obtained after incubating transfected cells with human Fc only (~10%, Figures 4E–E‴,G). Ncam1a binds weakly to Fgfr1a-IIIb only, its binding coefficient to the other isoforms does not exceed background levels (Supplementary Figure S5). The interaction concluded from this analysis was further confirmed by fluorescence intensity measurements. Cells transfected with membrane bound Fgfr1a isoforms showed a fourfold increase in fluorescence intensity compared to untransfected cells (data not shown). When analyzing the fluorescence intensity of soluble Ncam1b bound to the cells, we found that Fgfr1a-IIIb positive cells show 80% higher fluorescence intensities than untransfected cells (Figure 4J). Fgfr1a-IIIc transfected cells show a lower fluorescence intensity for Ncam1b, as well as cells expressing a receptor isoform without exon 7. This suggests that Ncam1b has a higher affinity for Fgfr1a-IIIb than for the other splice variants. Ncam1a in general has a lower affinity for all Fgfr1a isoforms (Supplementary Figure S5J).

As cell overlay assays do not clearly indicate in which orientation, *cis* or *trans*, a soluble ligand interacts with a membrane-tethered binding partner, we next performed bead aggregation assays. Magenta fluorescent ProteinA beads were coated with either Ncam1a-Fc or Ncam1b-Fc, and green fluorescent ProteinA beads were coated with either Fgfr1a-IIIb-Fc or Fgfr1a–IIIc-Fc. By incubating different combinations of the beads we found large clusters of Ncam1b-coated beads (Figures 4H,I) as well as of Ncam1a-coated beads (Supplementary Figures S5H,I). Clusters of Ncam1b-coated beads incorporate small clusters of Fgfr1a-IIIb-coupled beads (Figure 4H and Supplementary Figure S6A–A″) and to a weaker extent small clusters of Fgfr1a-IIIc-coupled beads (Figure 4I and Supplementary Figures S5B–B″). Clusters of Ncam1a-coupled beads rarely incorporate neither Fgfr1a-IIIb nor Fgfr1-IIIc coupled beads (Supplementary Figures S5H,I and Supplementary Figures S6C–D″).

Finally, we performed co-immunoprecipitations of Fgfr1a-FLAG and Ncam1-His co-expressed as membrane-bound proteins in *HEK293* cells. Immunoprecipitates of either Ncam1b or Ncam1a contain both receptor splice variants, Fgfr1a-IIIb or Fgfr1a-IIIc, (Supplementary Figure S5), supporting the interactions concluded from the experiments described above.

In combination our results indicate that Ncam1b binds to Fgfr1a in *trans in vitro*, with the isoform Fgfr1a-IIIb being a stronger binding partner than Fgfr1a-IIIc. Ncam1a binds Fgfr1a only weakly. This may result from the lack of a CAM Homology Domain (CHD) in Ncam1a (Supplementary Figure S7, see also discussion).

### A Knockdown of *fgfr1a* Partially Phenocopies the *ncam1b-*Knockdown

**Figure 5:**
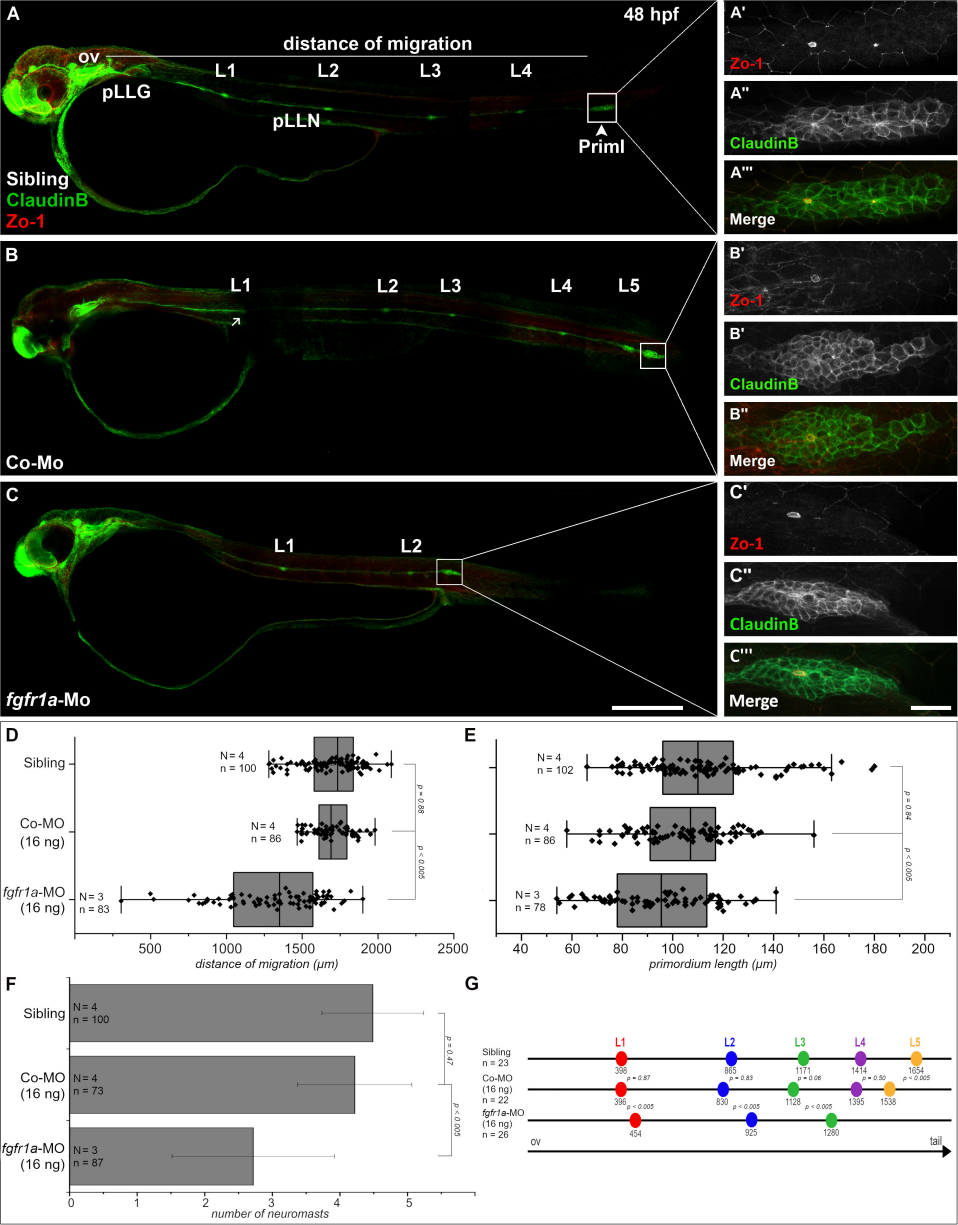
Fgfr1a is important for posterior lateral line development. Lateral views of 48 hpf *Tg(ClaudinB::lynGFP)* (green) embryos. **(A,B)** In siblings and control-morpholino injected embryos Priml has reached the tip of the tail and deposited 5 neuromasts. **(C)** *fgfr1a-*knockdown embryos show a delayed migration of primordia and a reduced number of neuromasts. **(A′-C′)** Apical constriction, visualized by staining of Zo-1, occurs under all conditions. Quantification of **(D)** migration distance, **(E)** primordium length, **(F)** number and **(G)** spacing of neuromasts, respectively. Error bars in bar chart represent standard deviation. Scale bars **(A,B)** 200 μm, **(A′-C″)** 20 μm. Abbreviations: Co-Mo, Control-Morpholino; L1-6, lateral (pro)neuromast; pLLGg, lateral line ganglion; pLLN, lateral line nerve; ov, otic vesicle; PrimI, primary posterior primordium.

Since Ncam1b affects Fgfr1a signaling, as judged by reduced *erm* expression, we expected that knocking down *fgfr1a* should have similar effects on pLLS development as knocking down *ncam1b*. In fact, we observed a reduced migration distance of the primordium after a morpholino-knockdown of *fgfr1a* that targets all isoforms (Figures 5C,D). At 48 hpf, *fgfr1a-*knockdown primordia have covered about two thirds of the migration path whereas primordia of uninjected siblings or control-morpholino injected embryos have already reached the tip of the tail. In addition, the size of the primordium is significantly reduced (Figures 5A′-C‴,E). A third effect of the *fgfr1a-*knockdown is a reduced number of neuromasts (Figures 5A–C,F). As a reduction of neuromast numbers may result from defects in proneuromast development, we studied rosette formation during the initial phase of primordium migration, at 24 hpf. After *fgfr1a-*knockdown we find a strongly reduced Zo-1 staining in the primordium; Zo-1 is a *zonula occludens* protein which marks apical constriction sites in nascent proneuromasts (Supplementary Figure S8). Accordingly the knockdown of *fgfr1a* affects proneuromast formation, similar as has been observed in studies using Fgfr inhibitors (Lecaudey et al., 2008; Ernst et al., 2012).

We also measured neuromast spacing at 48 hpf (Figure 5G). In uninjected siblings the first proneuromast is deposited after approximately 400 μm of primordium migration, whereas primordia of *fgfr1a*-morphants deposit their first proneuromast after approximately 450 μm. These experiments demonstrate that Fgfr1a signaling is crucial for the formation of proneuromasts and for the timing of their deposition. In general, the knockdown of *fgfr1a* affects pLLS development in a similar way, yet not as strongly as the knockdown of *ncam1b*. It cannot be excluded that this is due to different efficacies of the used morpholinos. *One* may however also assume that the knockdown of *ncam1b*, besides attenuating Fgfr1a signaling, weakens Ncam1b-dependend cell adhesion in the primordium. This could explain the rapid disintegration of rosettes in the morphants (Supplementary Video 4).

### Ncam1b Impairs Expression of Cxcr7b

In addition to primordial cell proliferation and proneuromast deposition, the knockdown of *ncam1b* affects the migration of the primordium. By the time the primordium of uninjected siblings has reached the tip of the tail, the morphant primordium has only covered about half the distance (Figure 1E). In some cases, we also found primordia which leave their path and migrate backward (Supplementary Figures S9B–E, Supplementary Videos 5, 6). Other primordia left their path along the horizontal myoseptum, migrated to the yolk border, returned to their original path, and continued migration toward the tail (Supplementary Video 7).

**Figure 6:**
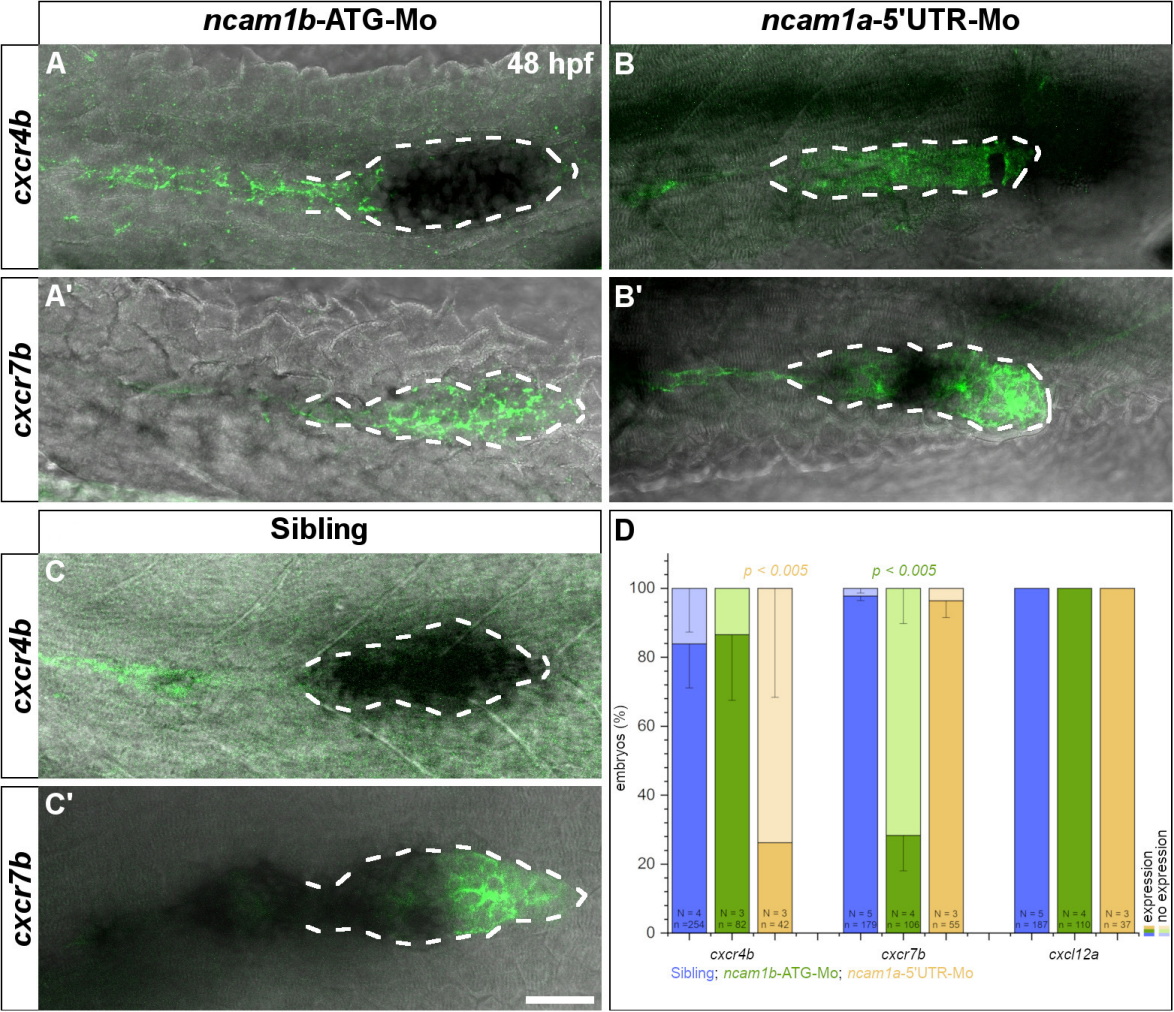
Expression of chemokine receptor *cxcr7b* is affected by knockdown of *ncam1b*. *Tg(ClaudinB::lynGFP)* embryos (48 hpf) were immunostained for GFP (green). *In situ* hybridizations of probes detecting *cxcr4b* (complete PrimI) and *cxcr7b* (Trailing-Zone), were visualized by NBT/BCIP staining. Primordia outlines were surrounded with a dotted line for better visibility. (**A,A′**) Expression of *cxcr4b* is not affected in *ncam1b*-morphants, whereas expression of *cxcr7b* is markedly reduced. (**B,B′**) In *ncam1a*-morphants *cxcr7b* is present, whereas *cxcr4b* expression is reduced. (**C,C′**) In siblings, *cxcr7b* is robustly expressed in the Trailing-Zone. (**D**) Drastically reduced *cxcr7b* expression after knockdown of *ncam1b*. Error bars represent standard deviation. X^2^-test with *ns* = *p* ≥ *0.05*. Scale bar 20 μm.

The pLLS primordium migrates along the horizontal myoseptum following a path which is delineated by the homogenous expression of chemokine Cxcl12a (Sdf1). Directionality is regulated by polarization of the primordium primarily through asymmetric expression of the chemokine clearance receptor Cxcr7b (Boldajipour et al., 2008; Aman et al., 2011; Donà et al., 2013; Venkiteswaran et al., 2013; Lau et al., 2020). While the expression patterns of the second chemokine receptor, *cxcr4b*, are unaffected by morpholino-knockdown of *ncam1b* (Figure 6A, compare to 6C), we found the amount of *cxcr7b* mRNA to be markedly reduced (Figure 6A′, compare to 6C′, and 6D). The morpholino-knockdown of *ncam1a* does not affect the expression of *cxcr7b* (Figure 6B′); we find a reduction of *cxcr4b*, which we cannot explain (Figure 6D). Combining the above results, it is tempting to speculate that Ncam1b directs primordial migration by regulating *cxcr7b* expression.

## Discussion

### Ncam1b Affects Development of the Zebrafish Lateral Line System

The formation of the posterior lateral line system (pLLS) strongly depends on cell adhesion molecules. So far, calcium-dependent cell adhesion molecules like cadherins have been reported to assure cell type-specific adhesion in the neuromasts (Matsuda and Chitnis, 2010; Colak-Champollion et al., 2019). In the present study we describe the calcium-independent neural cell adhesion molecule Ncam1b as part of the Wnt/Fgfr1 interaction network which controls pLLS development in zebrafish (Aman and Piotrowski, 2008; Lecaudey et al., 2008; Aman et al., 2011; Breau et al., 2012; Ernst et al., 2012; Lush and Piotrowski, 2014; Venero Galanternik et al., 2015). Morpholino-knockdowns of *ncam1b* (i) cause a size reduction of the pLLS primordium, (ii) reduce the number and the stability of deposited proneuromasts, and (iii) affect migration of the primordium (Figure 1).

### Ncam1b Regulates Cell Proliferation in a Fgfr1a-Dependent Manner

The size of the pLLS primordium depends on cell proliferation which occurs in both, Leading- and Trailing-Zones (Figure 2) (Nogare and Chitnis, 2017). In *ncam1b*-morphants, the cell division rate is markedly reduced as our BrdU assays show (Figure 2). In principle this could suggest a role of Ncam1b in either or both signaling pathways controlling cell division within the primordium, namely the Wnt and Fgfr1a pathways.

A major regulator of primordial cell proliferation is the Wnt-target gene product Lef1 (Lecaudey et al., 2008; Gamba et al., 2010; Valdivia et al., 2011; Breau et al., 2013; Agarwala et al., 2015). We found, however, that *lef1* expression in the Leading-Zone is unaltered in *ncam1b*-morphants (Figure 3A) while proliferation is drastically reduced. Accordingly, Lef1 is not sufficient for inducing primordial cell proliferation. This is supported by studies in *lef1*-mutants, which still show proneuromast formation and deposition, indicating that besides Lef1 other key regulators for primordial cell proliferation must exist (McGraw et al., 2011, 2014). Ncam1b is not expressed in the Leading-Zone, however, it shares expression domains with Fgfr1a in the Trailing-Zone (Figures 1C′, 3B″). In addition, the expression of the Fgfr1 target gene *erm* is inhibited in *ncam1b-*morphants (Figure 3C) and a knockdown of *erm* results in a reduced proliferation in the Trailing-Zone (Supplementary Figure S8). This suggests that an interaction of Ncam1b with Fgfr1a might be involved in regulating cell proliferation. This is supported by two studies showing that an inhibition of the Fgfr1a receptor strongly reduces primordial cell proliferation in zebrafish (Aman et al., 2011; McGraw et al., 2014). In mature neuromasts proliferation and regenerative cell proliferation are also promoted via FGF signaling and not directly through the Wnt pathway (Tang et al., 2019). A role of Erm (Etv5b) in proliferation was recently shown in murine embryonic stem cells (Akagi et al., 2015).

We here demonstrate a direct interaction between zebrafish Ncam1b and Fgfr1a by cell overlay, bead aggregation and co-immunoprecipitation assays. Binding crucially depends on the C-terminal part of the 3rd Ig-domain of Fgfr1a. We observed a much stronger interaction with isoform Fgfr1a-IIIb (Figures 4A–B‴,G,H,J) than with isoform Fgfr1a-IIIc (Figures 4C–D‴,G,I,J), which differ in the 3rd Ig-domain as a result of alternative splicing. Fgfr1a-IIIa, which lacks the C-terminal part of the 3rd Ig-domain, does not bind Ncam1b (Figures 4E–E‴,G,J).

In contrast to Ncam1b, Ncam1a shows almost no affinity to either Fgfr1a-IIIb or Fgfr1a-IIIc (Supplementary Figure S5). To find molecular signatures which account for this difference, we searched the amino acid sequences of both zebrafish Ncam1 paralogs for domains which have been previously identified as putative sites for an interaction with FGFR1 in rats and mice (Saffell et al., 1994; Williams et al., 1994a; Kiselyov et al., 2003; Kochoyan et al., 2008). We found no differences in the *FG Loop* interaction sequences in the FN-domains of zebrafish Ncam1a and Ncam1b; likewise, their counterparts in the 2nd and 3rd Ig-domain of the zebrafish Fgfr1a isoforms are preserved (Supplementary Figure S7). Besides these direct NCAM1-FGFR1 interaction sites, the *CAM Homology Domain* (CHD) in the 4th Ig-domain of NCAM1 has been identified as an important binding site. This domain is supposed to be involved in NCAM1 homophilic interactions required for triggering the formation of complexes with FGFR1 (Williams et al., 1994a; Doherty and Walsh, 1996; Anderson et al., 2005; Kochoyan et al., 2008). A removal of this domain inhibits FGFR1 signaling (Williams et al., 1994a). Whereas, zebrafish Ncam1b contains a CHD, Ncam1a lacks this sequence (Supplementary Figure S7C). This may account for its severely decreased affinity for Fgfr1a.

We find that a knockdown of *fgfr1a* partially phenocopies the *ncam1b*-knockdown (Figure 5 and Supplementary Figure S8). This finding alone does not directly support a role of Ncam1b in Fgfr1a-mediated signaling. However, the facts that (i) Ncam1b affects expression of the Fgfr1a target gene *erm* and (ii) both knockdowns have similar effects strongly argue for this interaction. We observed that slightly more proneuromasts are formed and deposited in *fgfr1a*-morphants than in *ncam1b*-morphants. This may be attributed to a cell-adhesive role of Ncam1b in stabilizing neuromasts once they are formed (see below).

**Figure 7:**
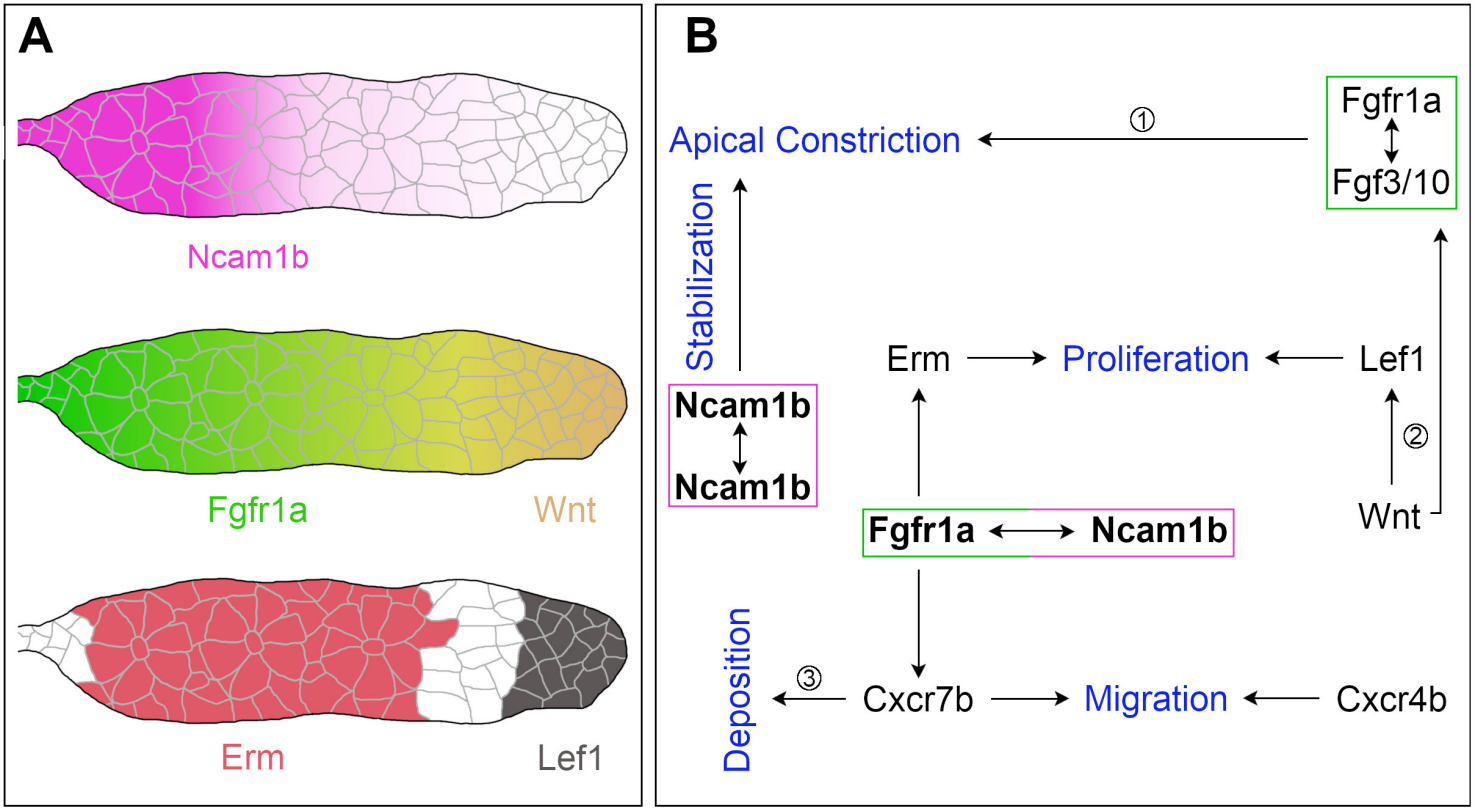
The function of Ncam1b during posterior lateral line development. **(A)** Expression patterns of Ncam1b, Fgfr1a, Wnt, Erm and Lef1 in the pLLS primordium. **(B)** Interaction of Ncam1b with Fgfr1a results in activation of the transcription factor Erm, which is involved in cell proliferation in the Trailing-Zone of PrimI. The Ncam1b/Fgfr1a interaction also initiates the expression of the chemokine receptor Cxcr7b, which is necessary for directional migration of the PrimI and for proneuromast deposition. Wnt signaling controls proliferation in the Leading-Zone via Lef1. In addition, it triggers Fgf signaling in the Trailing-Zone, which initiates apical constriction during formation of proneuromasts. Proneuromasts are stabilized by homophilic Ncam1b binding in *trans*. (Data related ① to Lecaudey et al. (2008) ② Aman et al. (2011) ③ Aman and Piotrowski (2008).

In conclusion, our data suggest that Ncam1b acts as a non-canonical ligand for Fgfr1a which stimulates primordial cell proliferation by activating the downstream signal Erm (Figure 7). It is conceivable that the proposed Ncam1b-Fgfr1a signaling pathway regulates cell proliferation mainly in the Trailing-Zone, where both are expressed. Cell proliferation in the Leading-Zone is activated by the Wnt-Lef1 pathway (Gamba et al., 2010; Aman et al., 2011; Valdivia et al., 2011). Thus, we propose a mechanism in which both, Erm and Lef1, regulate mitosis in the primordium (Figure 7). Ncam1b/Fgfr1a/Erm are involved in the generation of cells, which differentiate toward a mechanosensory character, whereas Wnt/Lef1 aids the proliferation of mesenchymal cells.

### Ncam1b Is Involved in Proneuromast Formation and Stabilization

Besides cell proliferation and primordium size, proneuromast formation and deposition are affected in *ncam1b*-morphants. We find at most a single proneuromast being formed and deposited (Figure 1G). Precursors of proneuromasts are established once a group of cells has evaded the influence of Wnt signaling in the Leading-Zone and reached the Fgf signaling range in the Trailing-Zone. The Trailing-Zone is small in *ncam1b*-morphants due to a reduced overall size of the primordium and an increase of the Wnt signaling domain (Figure 3A). Accordingly, the number of proneuromasts is strongly reduced in *ncam1b*-morphants. The proneuromasts formed and deposited in *ncam1b*-morphants consistently disintegrate shortly after being released from the migrating primordium (Supplementary Video 4). A similar fragmentation phenotype has been reported in *notch* mutants, where it was attributed to a loss of cadherin-mediated adhesive interactions between prospective proneuromast cells (Matsuda and Chitnis, 2010). Accordingly, Ncam1b may stabilize the epithelial rosettes by strengthening the apical cohesiveness of the central hair and the surrounding support cells. This could be achieved through homophilic *trans* interactions, which constitute the canonical Ncam1 mode of action.

### Ncam1b Is Involved in Primordial Migration

The small primordium of *ncam1b*-morphants eventually stops its migration halfway through the embryo. This is similar to the effect Lecaudey et al. (2008) describe for Fgf signaling-inhibited embryos; primordium migration stops due to an inability to form and deposit proneuromasts.

In addition, some primordia in *ncam1b*-morphants stray from their way along the horizontal myoseptum (Supplementary Figure S9). They either later return to their normal path while others “*u-turn*” rostrally. This can be attributed to the reduced expression of *cxcr7b* (Figure 6A′). Directionality of primordial migration is a consequence of the polarized expression of Cxcr4b and Cxcr7b. Cxcr7b in the Trailing-Zone degrades the chemokine ligand Cxcl12a resulting in a posterior to anterior gradient of this guidance cue (Donà et al., 2013). The expression of Cxcr7b is restricted to the Trailing-Zone by Wnt-mediated inhibition in the Leading-Zone (Aman and Piotrowski, 2008; Breau et al., 2013) and it is downregulated after inhibiting Fgfr signaling (Aman and Piotrowski, 2008). We have shown here a direct link between Ncam1b and Fgfr1a signaling, which could explain the reduced expression of *cxcr7b* and primordial migration defects in *ncam1b*-morphants. A similar phenotype was described for mutants affecting expression of the chemokine Cxcl12a/Sdf1 (Haas and Gilmour, 2006).

## Conclusion

Our findings lead us to propose the following model (Figure 7): The neural cell adhesion molecule Ncam1b is expressed in the Trailing-Zone of the posterior lateral line primordium. It directly interacts with Fgfr1a to firstly induce expression of transcription factor Erm. Erm in turn activates cell proliferation in the Trailing-Zone. Proliferation in the Leading-Zone is controlled by the Wnt pathway via Lef1. Secondly, Ncam1b induces expression of the chemokine receptor Cxcr7b. This could either be due to direct Fgfr1a signaling (as indicated in Figure 7) or result from an Fgfr1a dependent inhibition of Wnt signaling, which in turn inhibits Cxcr7b expression. Besides being involved in generating the Cxcl12a gradient that directs primordial migration, Cxcr7b marks the Trailing-Zone in which proneuromasts slow down to eventually be deposited. Thirdly, homophilic binding of Ncam1b between neighboring cells stabilizes the newly formed proneuromasts. Ncam1a, in contrast, is not crucial for pLLS development. This supports a sub-functionalization of the two Ncam1 paralogs in zebrafish as previously reported by Langhauser et al. (2012). In summary, we here describe Ncam1b as a novel player in the complex feedback network which orchestrates the development of the pLLS; it affects primordial cell proliferation, collective cell migration, and deposition of sensory proneuromasts.

## Data Availability Statement

All datasets generated for this study are included in the manuscript. The raw data supporting the conclusions of this article will be made available by the authors after request.

## Ethics Statement

The animal study was reviewed and approved by Animal experimentation: Zebrafish husbandry and experimental work were performed in strict accordance with the recommendations in the German Animal Protection Standards and were approved by the government of Baden-Württemberg, Regierungspräsidium Karlsruhe, Germany (Aktenzeichen: 35-9185.64/BH KIT).

## Author Contributions

RD carried out the conceptualization and methodology of the study, carried out molecular lab work, validated the results, performed formal analysis, and processed images, drafted, reviewed and edited the manuscript, and funding acquisition. AL participated in validation, formal analysis, molecular lab work, and image processing. SH and KB contributed to molecular lab work and image processing. MB carried out conceptualization, reviewed and edited the manuscript, and acquired funding. JB was part in conceptualization, writing original draft, review, and editing. All authors gave final approval for publication and agree to be held accountable for the work performed therein. All authors contributed to the article and approved the submitted version.

## Conflict of Interest

The authors declare that the research was conducted in the absence of any commercial or financial relationships that could be construed as a potential conflict of interest.

## Supplementary Material

Supplementary Figure S1 Injection of various control morpholinos shows efficacy and paralog specificity of *ncam1b*-ATG Morpholino. **(A–C)** Neither injection of an unrelated standard control (Co-Mo) nor a mismatch-morpholino (partially targeting the ATG-region) alter the migration of the primordium **(A)**, its length **(B)** or the number of neuromasts **(C)**. Co-injection of *ncam1b*-ATG- and *p53*-morpholino does not rescue the *ncam1b*-phenotype, indicating that the observed effects are not caused by increased cell death. Injection of a *ncam1b*-morpholino targeting the 5′UTR phenocopies the knockdown caused by blocking the ATG-region. Error bars in **(C)** show standard deviation. **(D)** Western blot analysis of p53 at 4 hpf indicates that none of the used morpholinos affects expression of the apoptosis marker. Expression of the Ncam1b protein at 48 hpf is drastically reduced following injection of the knockdown morpholinos *ncam1b*-ATG and *ncam1b*-5′UTR. Injections of the unrelated control-morpholino and mismatch-morpholino do not affect Ncam1b expression. Anti-Tubulin stainings were used as loading controls.

Supplementary Figure S2 Morpholinos targeting different ncam1b-mRNA sites affect posterior lateral line (pLLS) development in the same manner. **(A–F)** Lateral views of *Tg(ClaudinB::lynGFP)* embryos at 48 hpf. **(A)** Lateral line primordium reaches the tip of tail at 48 hpf and deposited several neuromasts. Neither injection of an unrelated control-morpholino (Co-Mo) **(B)** nor of a mismatch morpholino **(C)** affects the development of the pLLS. **(D)** Knockdown of *ncam1b* by blocking the ATG-region results in a delayed migration of the smaller-sized primordium and a reduced number of deposited neuromasts. **(E)** Co-injection of *p53*-Mo does not rescue the phenotype induced by the *ncam1b*-ATG-Mo, indicating the *ncam1b*-ATG-Mo does not act by inducing unspecific p53 side-effects. **(F)** Blocking the 5′UTR of *ncam1b*-mRNA phenocopies the knockdown caused by blocking the ATG-region. Scale bar 200 μm.

Supplementary Figure S3 e*rm* expression is not restricted to the lateral line primordium, but also found in brain structures at 36 hpf. **(A)** Detection of *erm* within the forebrain, eye and the midbrain-hindbrain-boundary (MHB) as well as in PrimI. **(B)** *ncam1b*-morphants still express *erm* in the forebrain, eye and MHB but not in PrimI.

Supplementary Figure S4 Erm affects cell proliferation within the Trailing-Zone of primordia. Siblings or morpholino-injected *Tg(ClaudinB::lynGFP)* embryos were treated with BrdU at 36 hpf for 20 min, fixed and immunostained for BrdU (red), GFP (green) and DAPI (blue). **(A,A″)** BrdU incorporation is reduced in *ncam1b*-morphants, but obvious in **(B,B″)** uninjected siblings. **(C,D)** Quantification of BrdU-labeled cells within primordia. Cell proliferation is mainly reduced in the Trailing-Zone. Scale bar 20 μm.

Supplementary Figure S5 Ncam1a weakly interacts with different isoforms of Fgfr1a. *CHO-K1* cells were transfected either with complete coding sequences (CDS) of one of the three Fgfr1a-isoforms (green; IIIb, IIIIc, or w/o Ex7) or with Ncam1a (magenta). Transfected cells were incubated for 1 hr with either soluble Ncam1a or one of the Fgfr1a-isoforms, fixed and immunostained for His-, Fc-Tag, Actin (red) and DAPI (blue). **(A–B****‴****)** Ncam1a interacts weakly with Fgfr1a-IIIb irrespectively of which of the proteins is membrane-bound. **(C–E****‴****)** Binding of Ncam1a to Fgfr1a-IIIc or to Fgfr1a which lacks exon 7 could not be detected. **(F) Top**: immunoprecipitates obtained from lysates of cells expressing His-tagged Ncam1a or Ncam1b, respectively, and Flag-tagged Fgfr1a-IIIb or Fgfr1a-IIIc, respectively, as indicated. Both, Flag-tagged Fgfr1a-IIIb and Fgfr1a-IIIc co-precipitate with Ncam1a and Ncam1b (band density (Intensity Density) for untransfected cells: 61,969.7 ± 22,350.7 and for transfected cells: Ncam1b/Fgfr1a-IIIb: 170,493.3 ± 47,282.2; Ncam1b/Fgfr1a-IIIc: 137,674.0 ± 17,380.8; Ncam1a/Fgfr1a-IIIb: 89,512.7 ± 28,322.6; Ncam1a/Fgfr1a-IIIc: 117,447.0 ± 74,743.5). **Bottom**: immunoprecipitates obtained from lysates of cells expressing His-tagged Ncam1b and/or Flag-tagged Fgfr1a-IIIb as indicated. Flag-tagged Fgfr1a-IIIb is contained only in precipitates obtained from lysates of cells expressing both proteins, excluding the possibility of an unspecific background (band density (Intensity Density) for untransfected cells: 31,604 and for transfected cells: Ncam1b: 25,442; Fgfr1a-IIIb: 31,972; Ncam1b/Fgfr1a-IIIb: 202,895). **(G)** Quantification of the binding of Ncam1a to splice variants of Fgfr1a. Binding coefficient represents the percentage of cells expressing the membrane-bound binding partner that were co-labeled by antibodies against the soluble binding partner. Error bars represent standard deviations. (**H,I)** Ncam1a-coated beads have a low affinity for Fgfr1a-IIIb- or –IIIc-coated beads. **(J)** Measuring the fluorescence intensity of soluble Ncam1a-Fc shows a low binding affinity for membrane-bound Fgfr1a-IIIb, Fgfr1a-IIIc, or Fgfr1a without exon 7 in the cell overlay assay. Scale bar in (**E****‴**) represents 20 μm and in **(I)** 10 μm. CDS, coding sequence; ECD, extracellular domain.

Supplementary Figure S6 Ncam1b interacts with clustered Fgfr1a-IIIb. Fluorescently labeled ProteinA-Beads were either coated with Ncam1b, Ncam1a, Fgfr1a-IIIb, Fgfr1a-IIIc or, as a control, with human Fc (hFc). **(A–A″)** Ncam1b and Fgfr1a-IIIb interact in *trans*. Thereby Ncam1b enables clustering of Fgfr1a-IIIb. **(B–B″)** Besides homophilic interactions of Ncam1b, just few Fgfr1a-IIIc-coated beads were incorporated into clusters of Ncam1b-coated beads. **(C–C″)** Ncam1a and Fgfr1a-IIIb rarely interact. **(D–D″)** Ncam1a shows low affinity for Fgfr1a-IIIc. **(E–H″)** Beads coated with Ncam1b, Ncam1a, Fgfr1a-IIIb or Fgfr1a-IIIc do not interact with human Fc-coated beads. Scale bar represents 20 μm.

Supplementary Figure S7 Ncam1b and Fgfr1a share sequences that code for known protein-protein interaction domains, whereas Ncam1a lacks the CHD-NCAM1 sequence. **(A–C)** Sequences of Ncam1 homologs. **(D)** Sequences of Fgfr1 homologs. **(A)** The *FGF receptor activation motif* (FRM) is located in the 1st FN-domain of Ncam1a and Ncam1b. **(B)** Both Ncam1 paralogs express the highly conserved *FG Loop* (FGL) sequence. **(C)** Ncam1b contains a CAM Homology Domain, which is not found in Ncam1a. **(D)** The CAM Homology Domain in the 2nd Ig-Domain of FGFR1 is found in both zebrafish splice variants.

Supplementary Figure S8 Knockdown of *fgfr1a* affects the size of PrimI and early formation of proneuromasts. Embryos were immunostained for GFP (green) and Zo-1 (red) **(A)** Lateral view of PrimI which just starts its migration at 24 hpf. It is still connected to the lateral line ganglion (pLLG). **(A–A****‴****)** Zo-1 staining depicts the formation of two proneuromasts within PrimI. **(B)** Migration initiation of PrimI and **(B–B****‴****)** proneuromast formation are not affected by injection of the control-morpholino. **(C)** Primordia of *fgfr1a*-morphants are reduced in size but do not show migration defects at this early developmental stage. **(C–C****‴****)** Formation of proneuromast is reduced as shown by Zo-1 staining. **(D–F)** Quantification of migration distance, primordia size and proneuromast number within PrimI. Error bars in **(F)** represent standard deviation. Scale bar in **(C)** 50 μm and in **(C****‴****)** 20 μm.

Supplementary Figure S9 The knockdown of *ncam1b* results in severe abnormalities in primordium shape and migration. Lateral views of *Tg(ClaudinB::lynGFP)* primordia at 48 hpf. **(A)** Primordia of uninjected siblings have an elongated shape and a constant size, they migrate on a direct path to the tip of the tail. **(B–D)** Primordia of *ncam1b*-morphants are reduced in size and lack directional migration. They either stop migration prematurely or they leave the horizontal myoseptum, perform a *u-turn* and migrate backwards. **(E)** *Double-*knockdown of *ncam1a* and *ncam1b* phenocopies the *single-*knockdown of *ncam1b*. Scale bar represents 20 μm.

Supplementary Video 1 Migration of the primordium in wild type siblings.

Supplementary Video 2 Control-morpholino injected embryos show no altered phenotype of the pLLS.

Supplementary Video 3 A knockdown of *ncam1a* has weaker effects on primordium migration and proneuromast formation.

Supplementary Video 4 The primordium of *ncam1b*-morphants just reaches the end of the yolk extension.

Supplementary Video 5 Knockdown of *ncam1b* leads primordia to deviate from their path along the horizontal myoseptum.

Supplementary Video 6 Knockdown of both *ncam1* paralogs phenocopies *ncam1b*-single knockdowns.

Supplementary Video 7 Primordia of *ncam1b*-morphants can result in different migration abnormalities.
