# Supplementary figures and images for: Cell Proliferation and Collective Cell Migration During Zebrafish Lateral Line System Development Are Regulated by Ncam/Fgf-Receptor Interactions
Source: Front Cell Dev Biol. 2021 Jan 14;8:591011. doi: 10.3389/fcell.2020.591011 (PMC7841142; doi:10.3389/fcell.2020.591011)

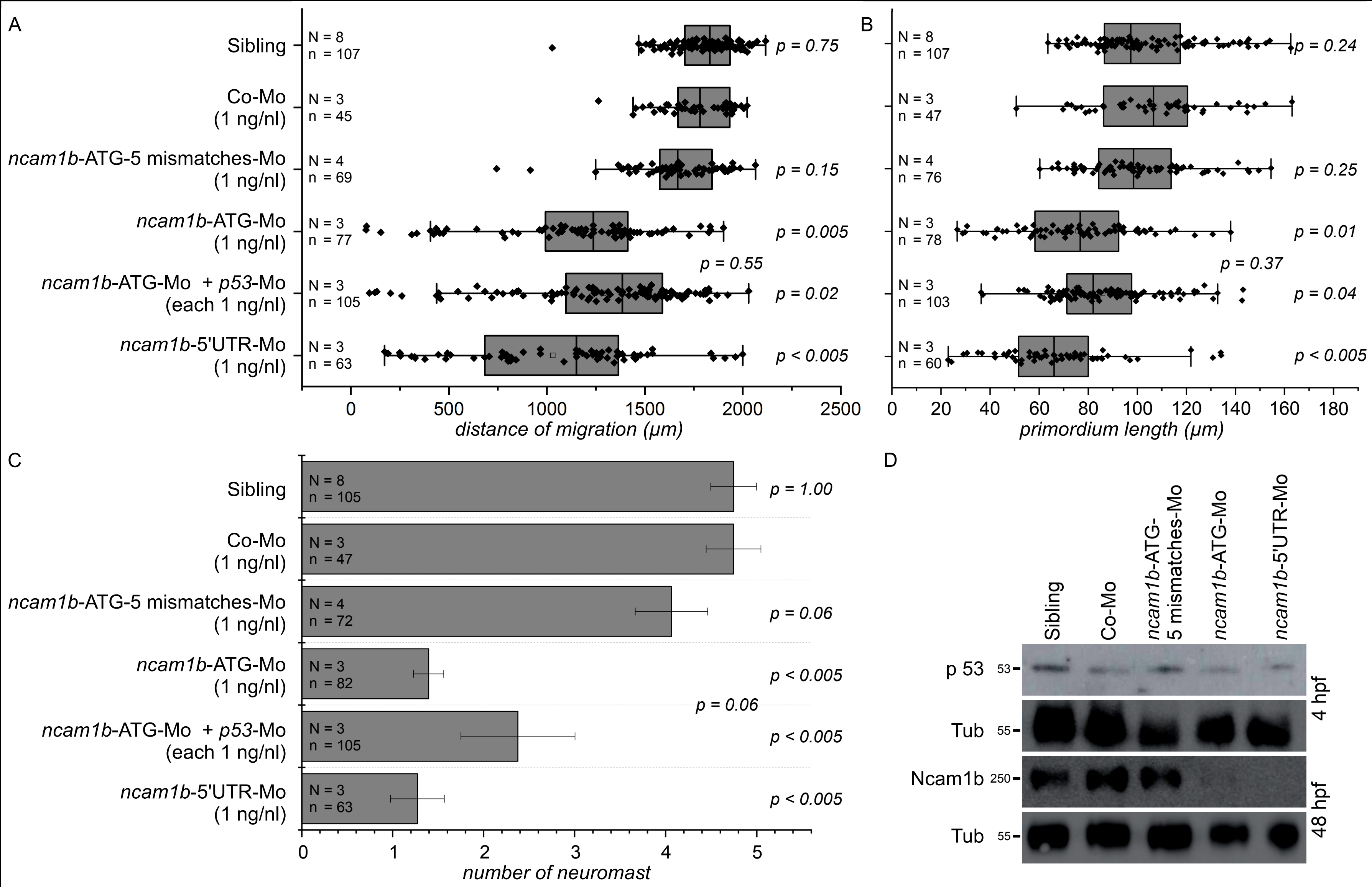

Supplement: Supplementary Figure S1 — Injection of various control morpholinos shows efficacy and paralog specificity of ncam1b-ATG Morpholino. (A–C) Neither injection of an unrelated standard control (Co-Mo) nor a mismatch-morpholino (partially targeting the ATG-region) alter the migration of the primordium (A), its length (B) or the number of neuromasts (C). Co-injection of ncam1b-ATG- and p53-morpholino does not rescue the ncam1b-phenotype, indicating that the observed effects are not caused by increased cell death. Injection of a ncam1b-morpholino targeting the 5′UTR phenocopies the knockdown caused by blocking the ATG-region. Error bars in (C) show standard deviation. (D) Western blot analysis of p53 at 4 hpf indicates that none of the used morpholinos affects expression of the apoptosis marker. Expression of the Ncam1b protein at 48 hpf is drastically reduced following injection of the knockdown morpholinos ncam1b-ATG and ncam1b-5′UTR. Injections of the unrelated control-morpholino and mismatch-morpholino do not affect Ncam1b expression. Anti-Tubulin stainings were used as loading controls. [file Image_1.pdf]

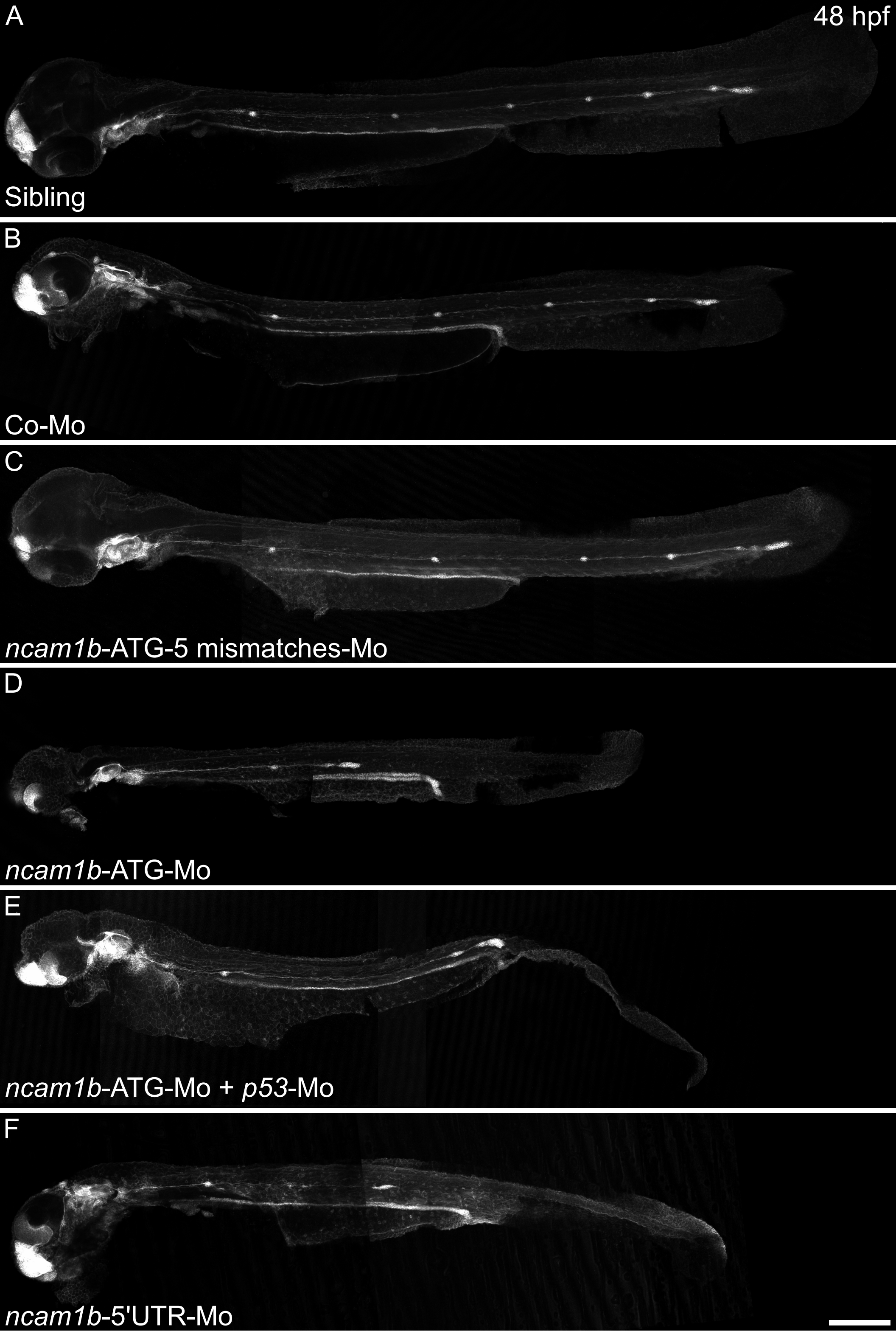

Supplement: Supplementary Figure S2 — Morpholinos targeting different ncam1b-mRNA sites affect posterior lateral line (pLLS) development in the same manner. (A–F) Lateral views of Tg(ClaudinB::lynGFP) embryos at 48 hpf. (A) Lateral line primordium reaches the tip of tail at 48 hpf and deposited several neuromasts. Neither injection of an unrelated control-morpholino (Co-Mo) (B) nor of a mismatch morpholino (C) affects the development of the pLLS. (D) Knockdown of ncam1b by blocking the ATG-region results in a delayed migration of the smaller-sized primordium and a reduced number of deposited neuromasts. (E) Co-injection of p53-Mo does not rescue the phenotype induced by the ncam1b-ATG-Mo, indicating the ncam1b-ATG-Mo does not act by inducing unspecific p53 side-effects. (F) Blocking the 5′UTR of ncam1b-mRNA phenocopies the knockdown caused by blocking the ATG-region. Scale bar 200 μm. [file Image_2.pdf]

**Sibling**

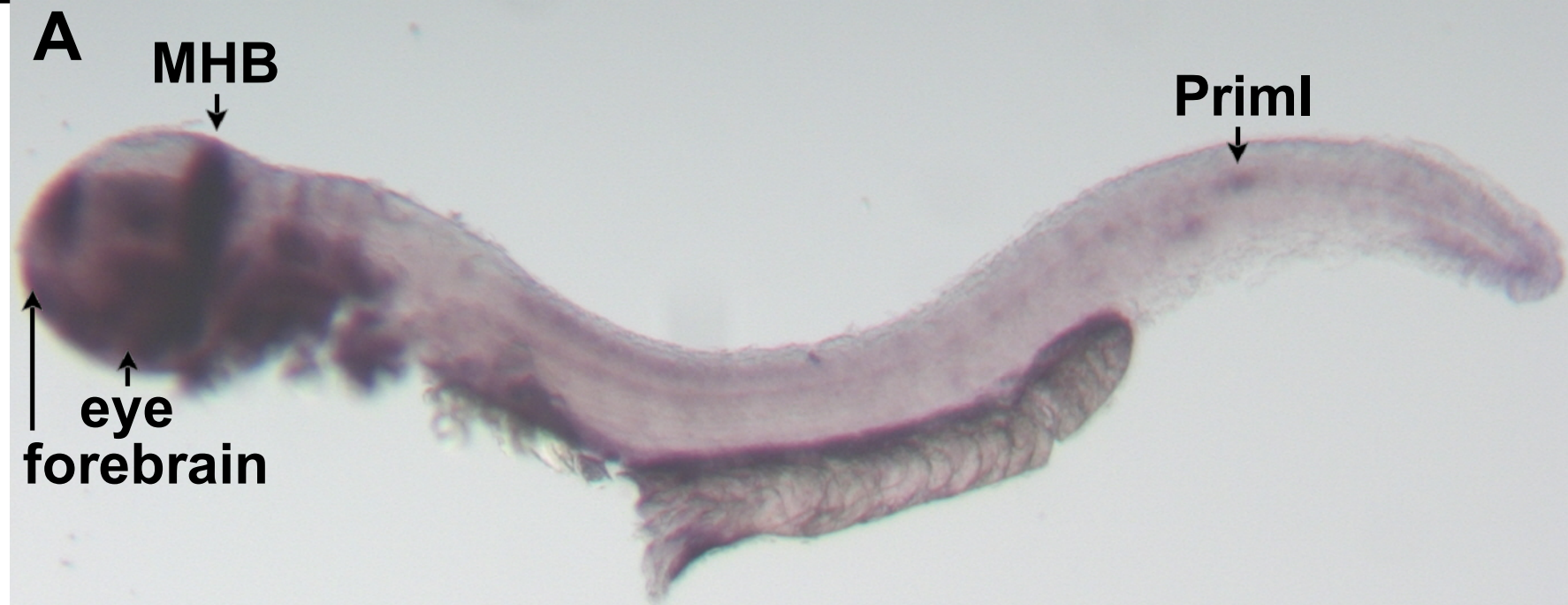

***ncam1b*-ATG-Mo**

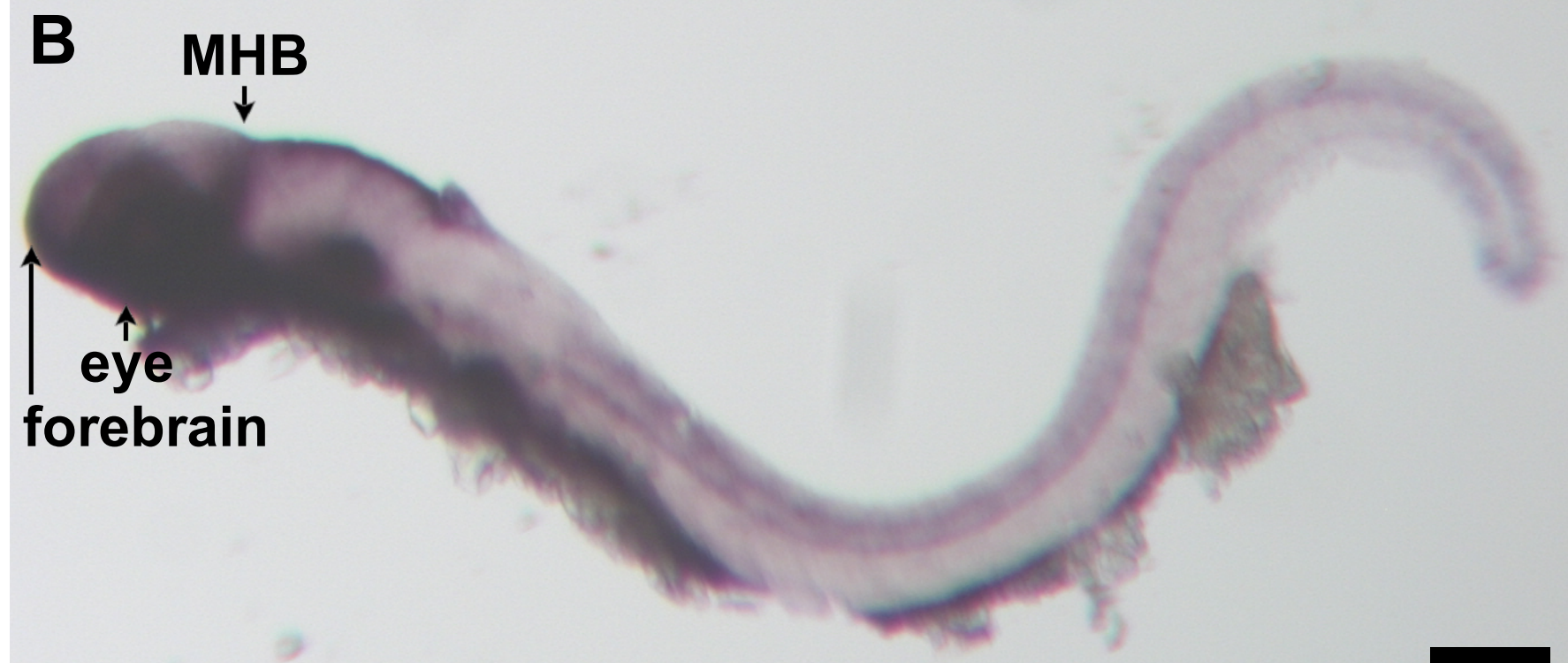

Supplement: Supplementary Figure S3 — erm expression is not restricted to the lateral line primordium, but also found in brain structures at 36 hpf. (A) Detection of erm within the forebrain, eye and the midbrain-hindbrain-boundary (MHB) as well as in PrimI. (B) ncam1b-morphants still express erm in the forebrain, eye and MHB but not in PrimI. [file Image_3.pdf]

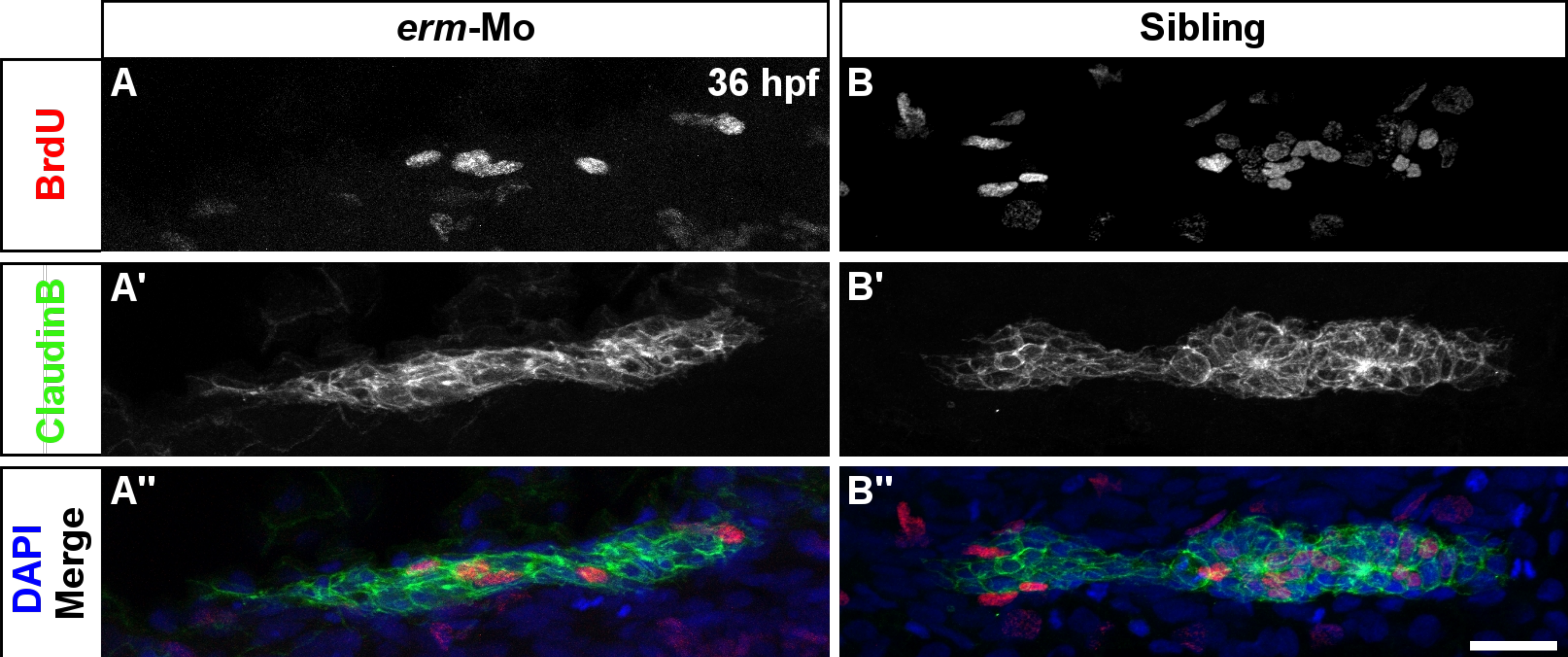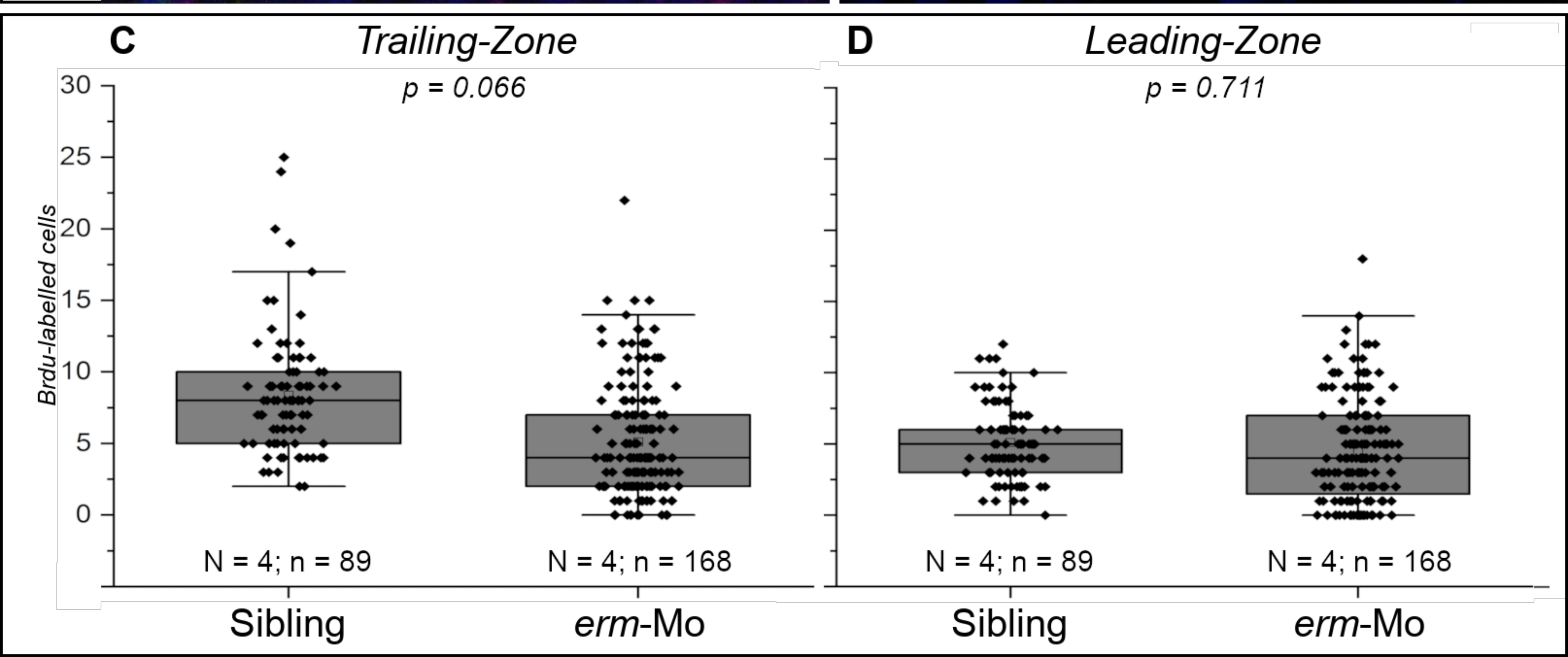

Supplement: Supplementary Figure S4 — Erm affects cell proliferation within the Trailing-Zone of primordia. Siblings or morpholino-injected Tg(ClaudinB::lynGFP) embryos were treated with BrdU at 36 hpf for 20 min, fixed and immunostained for BrdU (red), GFP (green) and DAPI (blue). (A,A″) BrdU incorporation is reduced in ncam1b-morphants, but obvious in (B,B″) uninjected siblings. (C,D) Quantification of BrdU-labeled cells within primordia. Cell proliferation is mainly reduced in the Trailing-Zone. Scale bar 20 μm. [file Image_4.pdf]

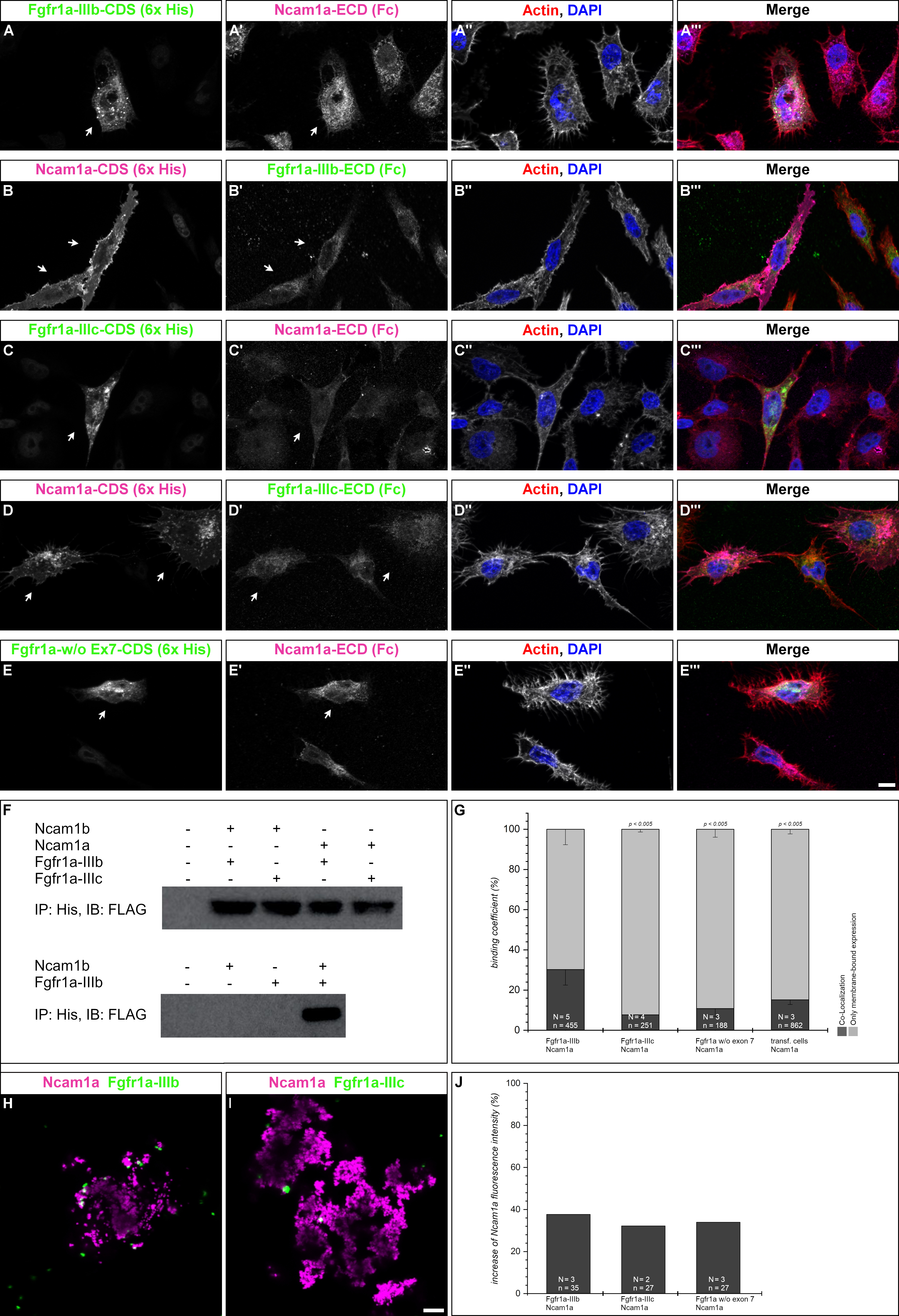

Supplement: Supplementary Figure S5 — Ncam1a weakly interacts with different isoforms of Fgfr1a. CHO-K1 cells were transfected either with complete coding sequences (CDS) of one of the three Fgfr1a-isoforms (green; IIIb, IIIIc, or w/o Ex7) or with Ncam1a (magenta). Transfected cells were incubated for 1 hr with either soluble Ncam1a or one of the Fgfr1a-isoforms, fixed and immunostained for His-, Fc-Tag, Actin (red) and DAPI (blue). (A–B‴) Ncam1a interacts weakly with Fgfr1a-IIIb irrespectively of which of the proteins is membrane-bound. (C–E‴) Binding of Ncam1a to Fgfr1a-IIIc or to Fgfr1a which lacks exon 7 could not be detected. (F) Top: immunoprecipitates obtained from lysates of cells expressing His-tagged Ncam1a or Ncam1b, respectively, and Flag-tagged Fgfr1a-IIIb or Fgfr1a-IIIc, respectively, as indicated. Both, Flag-tagged Fgfr1a-IIIb and Fgfr1a-IIIc co-precipitate with Ncam1a and Ncam1b (band density (Intensity Density) for untransfected cells: 61,969.7 ± 22,350.7 and for transfected cells: Ncam1b/Fgfr1a-IIIb: 170,493.3 ± 47,282.2; Ncam1b/Fgfr1a-IIIc: 137,674.0 ± 17,380.8; Ncam1a/Fgfr1a-IIIb: 89,512.7 ± 28,322.6; Ncam1a/Fgfr1a-IIIc: 117,447.0 ± 74,743.5). Bottom: immunoprecipitates obtained from lysates of cells expressing His-tagged Ncam1b and/or Flag-tagged Fgfr1a-IIIb as indicated. Flag-tagged Fgfr1a-IIIb is contained only in precipitates obtained from lysates of cells expressing both proteins, excluding the possibility of an unspecific background (band density (Intensity Density) for untransfected cells: 31,604 and for transfected cells: Ncam1b: 25,442; Fgfr1a-IIIb: 31,972; Ncam1b/Fgfr1a-IIIb: 202,895). (G) Quantification of the binding of Ncam1a to splice variants of Fgfr1a. Binding coefficient represents the percentage of cells expressing the membrane-bound binding partner that were co-labeled by antibodies against the soluble binding partner. Error bars represent standard deviations. (H,I) Ncam1a-coated beads have a low affinity for Fgfr1a-IIIb- or –IIIc-coated beads. (J) Measuring the fl [file Image_5.jpg]

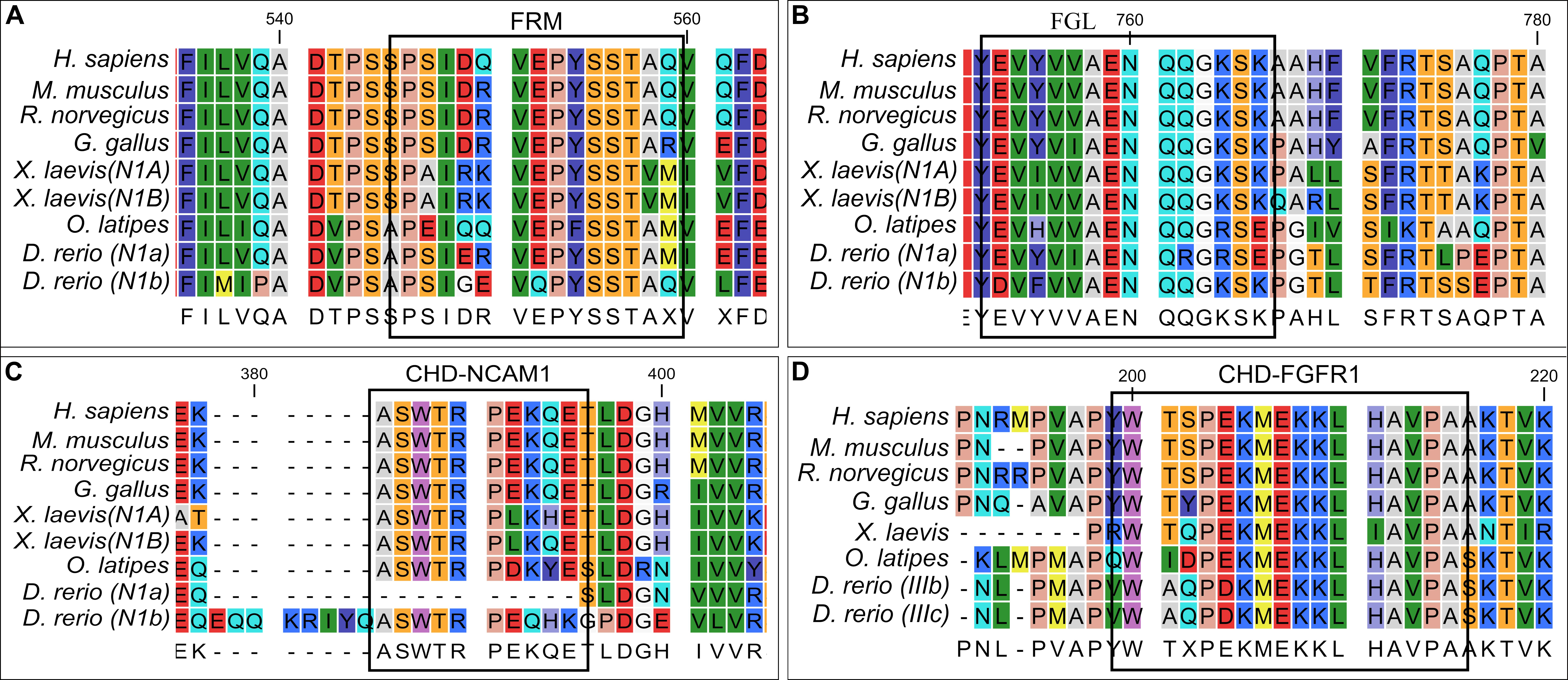

Supplement: Supplementary Figure S7 — Ncam1b and Fgfr1a share sequences that code for known protein-protein interaction domains, whereas Ncam1a lacks the CHD-NCAM1 sequence. (A–C) Sequences of Ncam1 homologs. (D) Sequences of Fgfr1 homologs. (A) The FGF receptor activation motif (FRM) is located in the 1st FN-domain of Ncam1a and Ncam1b. (B) Both Ncam1 paralogs express the highly conserved FG Loop (FGL) sequence. (C) Ncam1b contains a CAM Homology Domain, which is not found in Ncam1a. (D) The CAM Homology Domain in the 2nd Ig-Domain of FGFR1 is found in both zebrafish splice variants. [file Image_7.jpg]

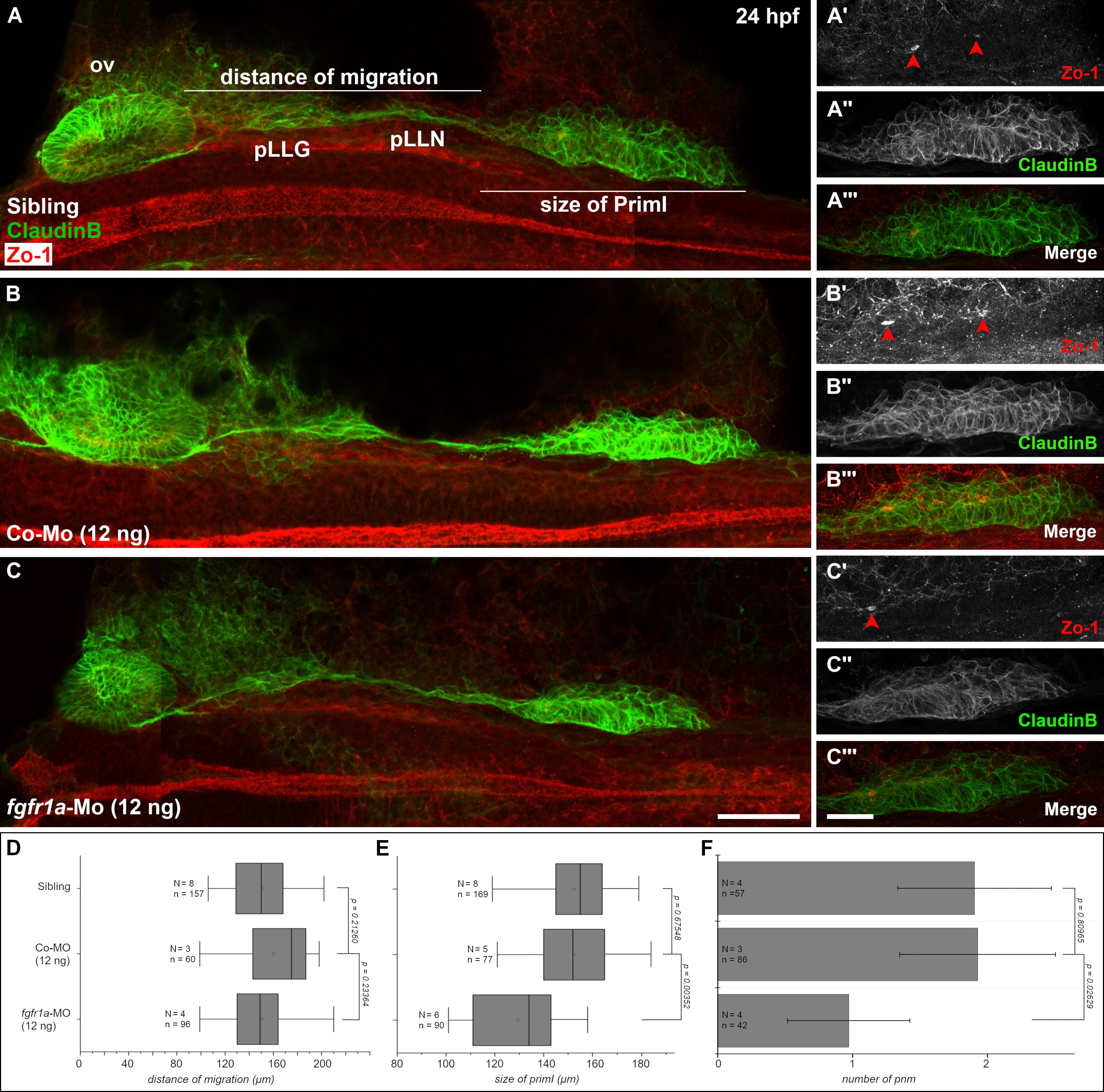

Supplement: Supplementary Figure S8 — Knockdown of fgfr1a affects the size of PrimI and early formation of proneuromasts. Embryos were immunostained for GFP (green) and Zo-1 (red) (A) Lateral view of PrimI which just starts its migration at 24 hpf. It is still connected to the lateral line ganglion (pLLG). (A–A‴) Zo-1 staining depicts the formation of two proneuromasts within PrimI. (B) Migration initiation of PrimI and (B–B‴) proneuromast formation are not affected by injection of the control-morpholino. (C) Primordia of fgfr1a-morphants are reduced in size but do not show migration defects at this early developmental stage. (C–C‴) Formation of proneuromast is reduced as shown by Zo-1 staining. (D–F) Quantification of migration distance, primordia size and proneuromast number within PrimI. Error bars in (F) represent standard deviation. Scale bar in (C) 50 μm and in (C‴) 20 μm. [file Image_8.jpg]

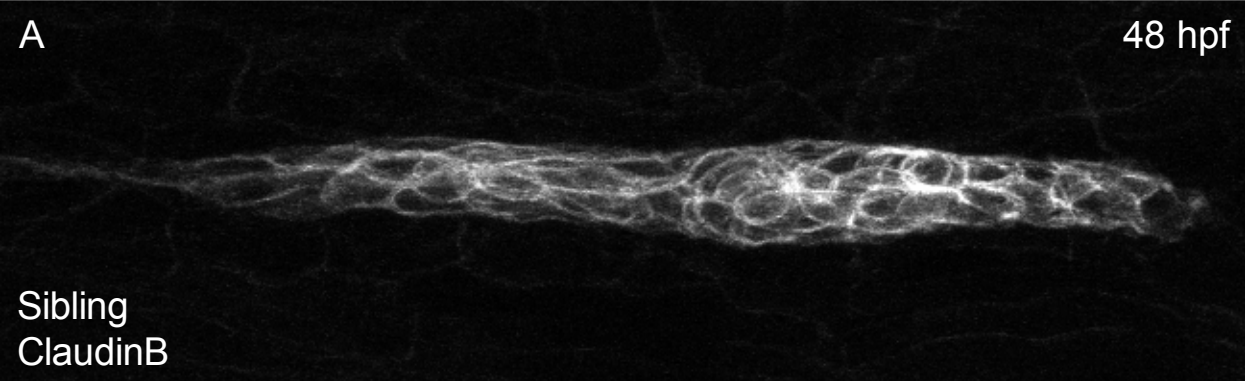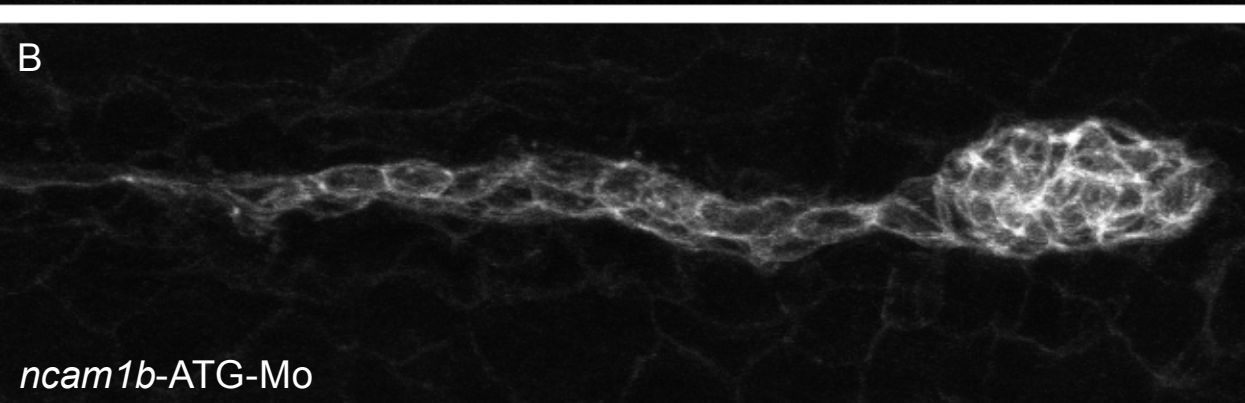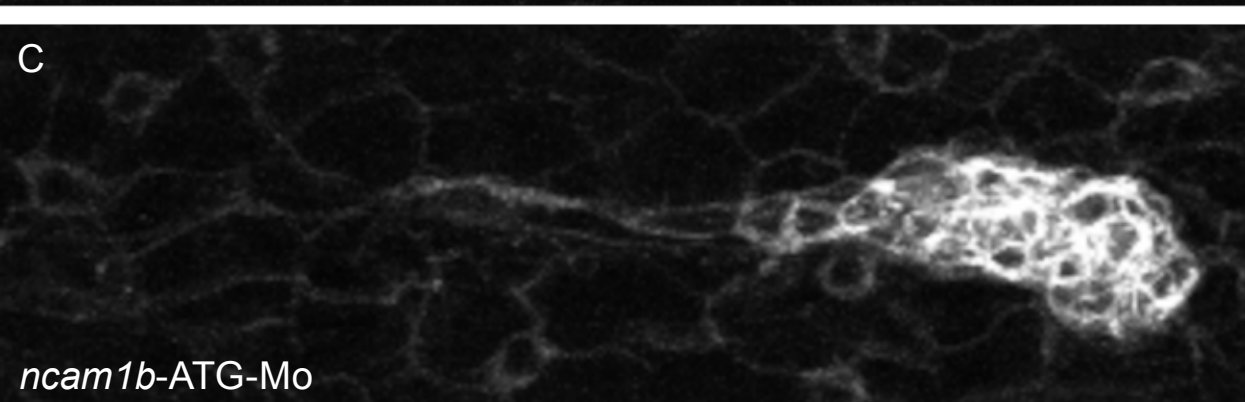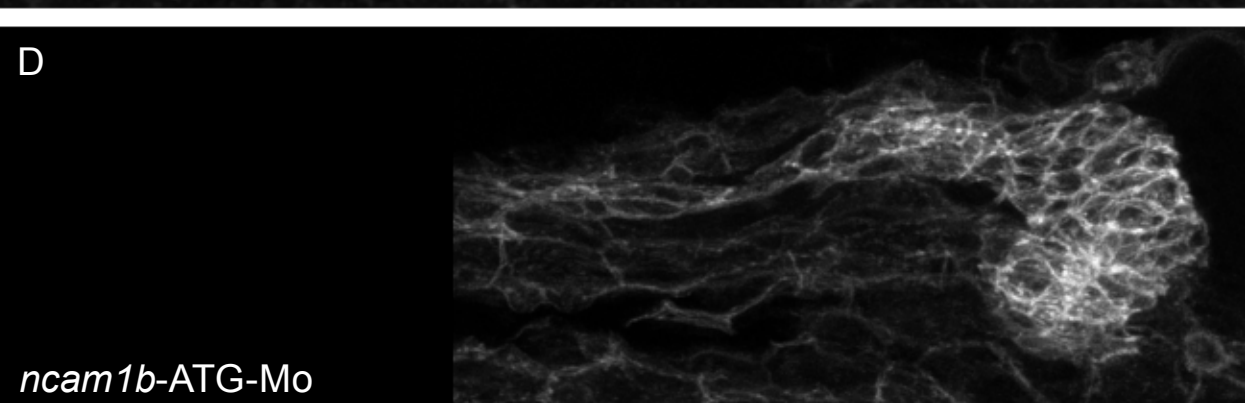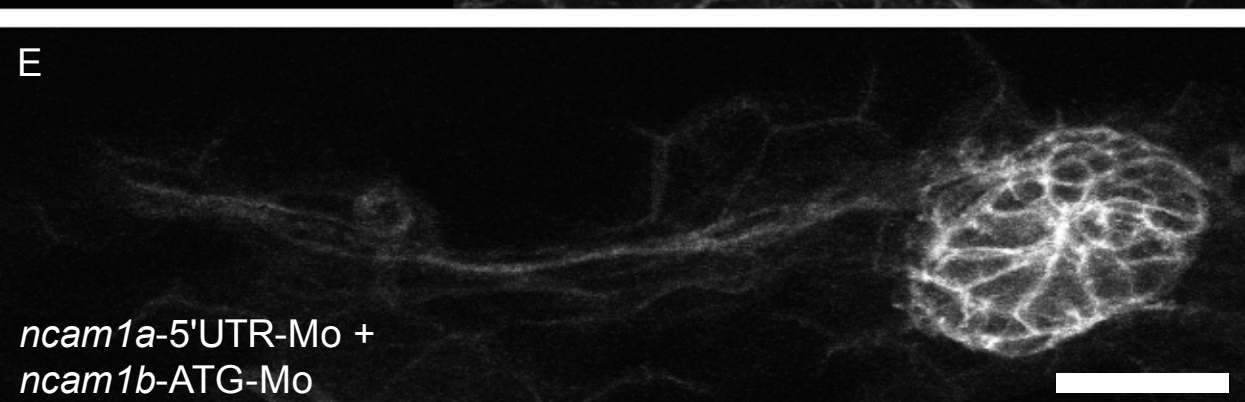

Supplement: Supplementary Figure S9 — The knockdown of ncam1b results in severe abnormalities in primordium shape and migration. Lateral views of Tg(ClaudinB::lynGFP) primordia at 48 hpf. (A) Primordia of uninjected siblings have an elongated shape and a constant size, they migrate on a direct path to the tip of the tail. (B–D) Primordia of ncam1b-morphants are reduced in size and lack directional migration. They either stop migration prematurely or they leave the horizontal myoseptum, perform a u-turn and migrate backwards. (E) Double-knockdown of ncam1a and ncam1b phenocopies the single-knockdown of ncam1b. Scale bar represents 20 μm. [file Image_9.pdf]
