# Supplementary material for: Cell Proliferation and Collective Cell Migration During Zebrafish Lateral Line System Development Are Regulated by Ncam/Fgf-Receptor Interactions
Source: Front Cell Dev Biol. 2021 Jan 14;8:591011. doi: 10.3389/fcell.2020.591011 (PMC7841142; doi:10.3389/fcell.2020.591011)
Supplement: Supplementary Figure S6 — Ncam1b interacts with clustered Fgfr1a-IIIb. Fluorescently labeled ProteinA-Beads were either coated with Ncam1b, Ncam1a, Fgfr1a-IIIb, Fgfr1a-IIIc or, as a control, with human Fc (hFc). (A–A″) Ncam1b and Fgfr1a-IIIb interact in trans. Thereby Ncam1b enables clustering of Fgfr1a-IIIb. (B–B″) Besides homophilic interactions of Ncam1b, just few Fgfr1a-IIIc-coated beads were incorporated into clusters of Ncam1b-coated beads. (C–C″) Ncam1a and Fgfr1a-IIIb rarely interact. (D–D″) Ncam1a shows low affinity for Fgfr1a-IIIc. (E–H″) Beads coated with Ncam1b, Ncam1a, Fgfr1a-IIIb or Fgfr1a-IIIc do not interact with human Fc-coated beads. Scale bar represents 20 μm. [file Image_6.pdf]

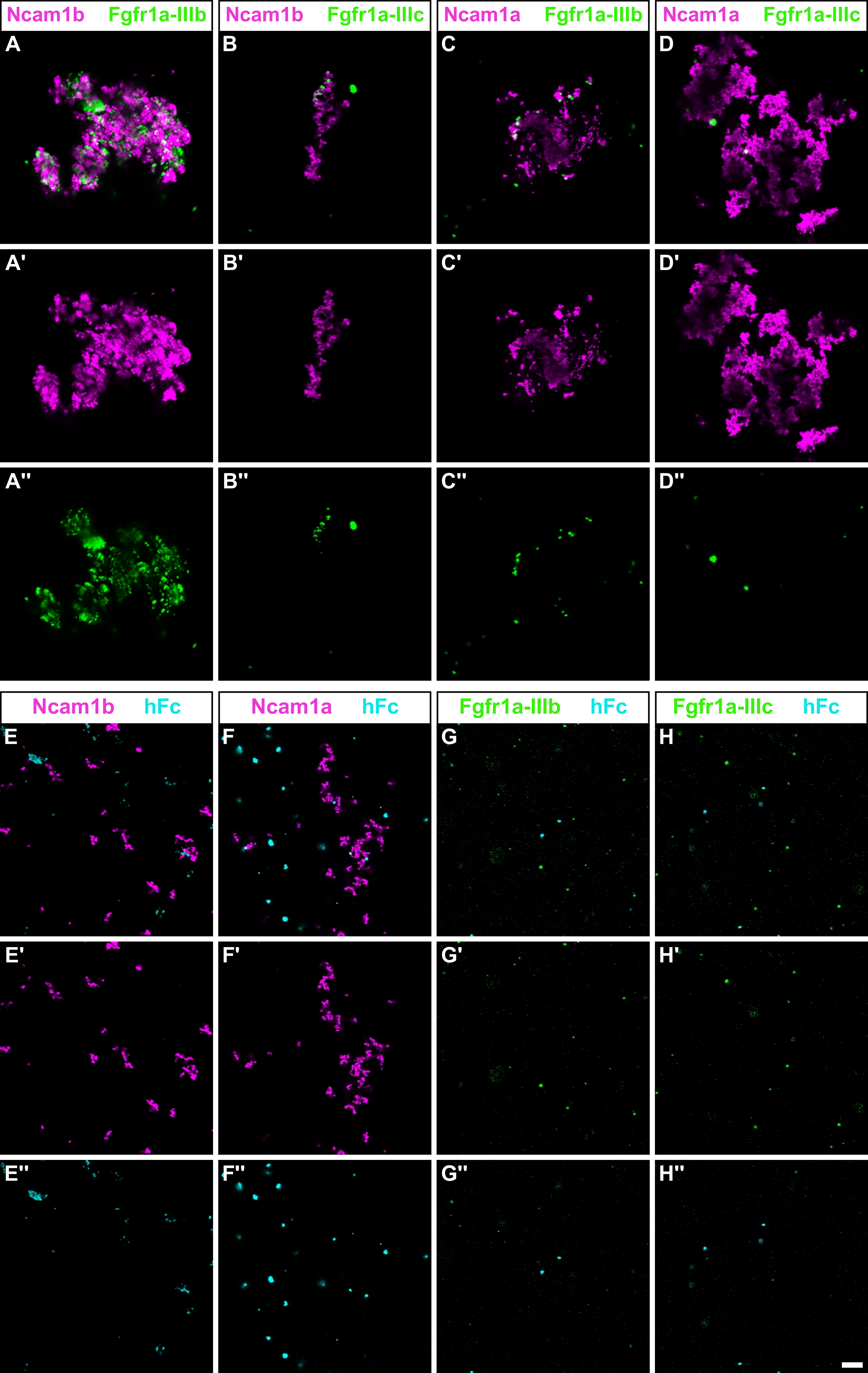

Ncam1b Fgfr1a-IIIb
Ncam1b Fgfr1a-IIIc
Ncam1a Fgfr1a-IIIb
Ncam1a Fgfr1a-IIIc
A
B
C
D
A'
B'
C'
D'
A''
B''
C''
D''
Ncam1b hFc
Ncam1a hFc
Fgfr1a-IIIb hFc
Fgfr1a-IIIc hFc
E
F
G
H
E'
F'
G'
H'
E''
F''
G''
H''
